# Moved by Observing the Love of Others: Kama Muta Evoked Through Media Fosters Humanization of Out-Groups

**DOI:** 10.3389/fpsyg.2020.01240

**Published:** 2020-06-24

**Authors:** Johanna K. Blomster Lyshol, Lotte Thomsen, Beate Seibt

**Affiliations:** ^1^Department of Psychology, University of Oslo, Oslo, Norway; ^2^Department of Political Science, Aarhus University, Aarhus, Denmark; ^3^Centro de Investigação e Intervenção Social (CIS-IUL), Instituto Universitário de Lisboa (ISCTE-IUL), Lisbon, Portugal

**Keywords:** kama muta, moved, humanization, media, out-group

## Abstract

People often view out-groups as less human than their in-group. Some media video content is *heart-warming* and leaves one feeling *touched* or *moved*. Recent research indicates that this reflects a positive social emotion, *kama muta*, which is evoked by a sudden increase in interpersonal closeness, specifically by the relational model of communal sharing. Because forming strong, close, and communal bonds exemplifies valued human qualities, and because other humans are our primary target partners of communal sharing, we predicted that feeling kama muta in response to observing communal sharing among out-group strangers would make people view out-groups as more human. In Study 1, we replicated a model obtained through a large exploratory preliminary study which indicated that videos depicting out-group members enacting communal sharing evoked kama muta and increased protagonist humanization. This, in turn, led to decreased blatant dehumanization of the entire out-group via perceived out-group warmth and motivation to develop a communal sharing relationship with the protagonist. The preregistered Study 2 further tested our model, demonstrating (1) that the relationship between protagonist humanization and kama muta is bidirectional such that baseline humanization of the protagonist also increases feelings of kama muta in response to acts of communal sharing; (2) that watching videos of communal sharing, as compared to funny videos, increased protagonist humanization; and (3) that kama muta videos, compared to funny videos, had an indirect effect on the reduction of out-group blatant dehumanization, which was mediated by protagonist humanization and out-group warmth.

## Introduction

“This clip was so beautiful, there were so many emotions and humor. Isak and Even just have their eyes on each other, it is so sweet! I have learnt so much from season 3 – that we just have to accept who we are, be open and not deal with things alone. The fact that we are first and foremost human – and not our sexuality, gender, disease, religion, etc.”

Anonymous comment from the official SKAM web page at the Norwegian Broadcasting Company (NRK)

The protagonists described in the quote above are vulnerable to stigmatization and to people seeing them as less than fully human. Isak and Even are a gay male couple and are lead characters in the Norwegian TV series “SKAM” about a group of friends at a high school in Oslo. The online comments’ sections of these videos reveal that viewers from all over the world overcome their own negative perceptions of gay men while watching SKAM. How does this happen? The series has attracted a lot of Norwegian and international attention due to its moving depictions of gay and minority teens in Norway (Krüger and Rustad, 2019). The comment above illustrates the emotional responses to the video clips, which evidently convey positive feelings about gay men. We posit that this reduction in negative perceptions is due to a specific emotional response that opens people to new social connections, an emotion which people often label as feeling moved.

Previous research has focused on the link between the negative emotion disgust and the perception of out-groups as less human than the in-group (e.g., Harris and Fiske, 2006; Bastian et al., 2013; Hodson et al., 2014; Dalsklev and Kunst, 2015). This line of research is based on the assumption that disgust inhibits people’s desire for social interactions, which leads them to dehumanize the person or group eliciting disgust (Sherman and Haidt, 2011). However, research on the link between positive emotions and the perception of out-groups as more human is scarce. The aim of the current paper is to close this gap by investigating the effect of an emotion which motivates social connections, namely, kama muta, on viewing out-groups as more human.

Here, we argue that one’s own emotional reaction in response to observing out-group members’ interactions should influence how human these persons, and possibly their groups, are perceived to be. These emotional reactions, in turn, will depend on the kinds of social relationships the persons are perceived to enact. These assumptions are based on theoretical frameworks stressing the crucial role of emotions for informing the pursuit of important evolved goals, such as the goal to cooperate with trustworthy others (Cosmides and Tooby, 2000; Fiske, 2002; Thomsen and Carey, 2013; Henrich, 2016).

Hence, we suggest that out-group members and their groups should be perceived as more human when viewers respond emotionally to the perception that out-group members are implementing extraordinary acts of communal sharing (e.g., taking care of each other and showing loving devotion) because such actions are an honest cue that they are benevolent cooperators. The emotional construct that fits best the assumed appraisal theme of perceiving such remarkable communal sharing is kama muta, a term recently introduced for a class of emotional experiences often labeled as being moved in English (Fiske et al., 2019). We therefore test whether the exposure to videos with such a theme leads to perceiving the protagonists and their groups as more human by evoking kama muta.

We start by outlining research on kama muta and explain how the relation between kama muta and humanization is plausible. Next, we delineate research on dehumanization and present findings showing that humanization is not necessarily the opposite of dehumanization. Lastly, we explicate the model in detail and summarize a preliminary study before presenting two preregistered studies which test our model.

### Kama Muta – A Positive Emotion Evoked by Witnessing Suddenly Intensified Communal Relations

Kama muta is a conceptualization of an emotion which people experience as a positive warm feeling. Kama muta is the Sanskrit term for *moved by love*. English speakers often use terms such as “being moved” or “touched” when they speak about feeling kama muta. In order to not only rely on vernacular labels when measuring and theorizing about kama muta (i.e., not commit the lexical fallacy, see Fiske, 2020) and to account for the cultural variability of kama muta, five components have been introduced as defining features of kama muta: (1) physiological signs, such as a warm feeling in the chest, tears, goosebumps, a lump in the throat, buoyancy, and/or exhilaration; (2) a positive valence; (3) vernacular labels such as moved, touched, and heart-warming; (4) the appraisal of a sudden intensification of a communal sharing relationship; and (5) that kama muta motivates people to strengthen or develop communal sharing relationships (Fiske et al., 2019). Strong kama muta typically includes several physiological signs, while a mild episode does not have to include any (Zickfeld et al., 2019a).

The central assumptions about the elicitors and effects of kama muta (points four and five above) are derived from Relational Models Theory (Fiske, 1991, 1992, 2004). Relational Models Theory proposes that humans coordinate and organize social interactions based on four universal relational models. The relational model of interest for kama muta is communal sharing (CS), which is characterized by interactions where people are perceived as being socially equivalent (Fiske, 2004) – as being the same kind. Therefore, when interacting according to the CS model, people feel one with others, through for example having the same identity, which orients their actions, motives, and thoughts to something they have in common – a common essence. Kindness, compassion, feelings of belonging, shared responsibility, and need-based sharing of resources are common in CS relations. CS relations are often operationalized as interpersonally close relationships (e.g., Schubert et al., 2018).

Kama muta is elicited when CS relations suddenly intensify (Fiske et al., 2019). CS intensification can be experienced because of something oneself or someone else initiates, or something that one observes, through videos for example (Fiske et al., 2019). Films and online videos that evoke kama muta often portray people intensifying their CS relationship. Examples of such videos portray reunions, proposals, and acts of kindness. Videos that have previously been used as stimuli evoking kama muta often have the following narrative: A communal sharing relation is first established, followed by an obstacle which endangers the communal sharing relation (evoking sadness instead), then ending with the affirmation and confirmation of the relation (Schubert et al., 2018). Because of a huge number of such videos that can be found online, some of them being extremely popular (Nelson-Field et al., 2013), we are interested in the psychological effects they have on their audience. Media scholars have shown the effectiveness of media to improve intergroup relations; Schiappa et al. (2005) found that viewing out-group members through media functions in the same manner as direct intergroup contact (i.e., developing close bonds with protagonists), which has proven to be effective in improving intergroup relations (Pettigrew and Tropp, 2006). The effect of kama muta is to motivate increased affective devotion and moral commitment to CS relationships (Fiske et al., 2017). Indeed, feeling kama muta has been shown to increase communal feelings toward the observed target whom experienced CS intensification (Zickfeld, 2015). Thus, we postulate that feeling kama muta from viewing out-group members portraying acts of communal sharing is a form of parasocial contact, which we predict will increase the perceived humanness of the protagonists and their groups.

The validity of both the kama muta construct and the KAMMUS, a multidimensional measure of kama muta, has been previously demonstrated in a cross-cultural study (Zickfeld et al., 2019a). Across 19 countries, in 5 continents and 15 languages the physiological signs, positive valence, vernacular labels, appraisals of sudden intensification of a CS relationship, and communal motivations were highly correlated. In addition, kama muta was shown to be different from sadness, awe, and amusement (Zickfeld et al., 2019a). Kama muta has also been shown to be reliably related to the trait measure of Empathic Concern (Zickfeld et al., 2017, 2019a), which assesses people’s disposition of feeling compassion toward others in need (Davis, 1980, 1983). This relationship also holds for kama muta being elicited by CS intensifications not involving needy individuals, such as proposal scenes, and episodes not involving empathic identification with others, such as receiving a Valentine’s surprise. Therefore, Zickfeld et al. (2017) propose that the trait of empathic concern predicts responses across a broader array of emotional scenes than previously assumed and that “the state of empathic concern is a specific case of experiencing the emotion kama muta when perceiving others in need” (Zickfeld et al., 2017, p. 4).

In sum, kama muta is a cross-culturally validated emotion, which is evoked by a sudden intensification of a communal sharing relation. In this paper, we specifically test the effect of kama muta evoked through parasocial contact on perceived humanness. We suggest that kama muta will increase perceived humanness because it signals a propitious opportunity for a new or renewed communal sharing relationship with a trustworthy, cooperative, sensitive, loving, and caring human being.

### Dehumanization and Humanization

Dehumanization is the process of not including people into the human category. Members of this human category share an essence, which connects all humans and differentiates us from other species (Leyens et al., 2000). Dehumanizing out-groups, i.e., perceiving them as not fully human, is a fairly common phenomenon (Haslam and Loughnan, 2014; Haslam and Stratemeyer, 2016). Disgust is closely related to dehumanization. This is because many disgust elicitors remind people of the fact that humans are not much different from animals (Rozin et al., 2008). Indeed, studies have shown that dehumanization is associated with disgust elicited by reading about unhygienic living conditions of Roma (Dalsklev and Kunst, 2015), violent or sexual crimes (Bastian et al., 2013), or refugees being immoral cheaters (Esses et al., 2012). Folk conceptions of dehumanization describe people as lacking culture and being irrational, crude, savage, amoral, and primitive (Demoulin et al., 2004; Haslam, 2006; Bain et al., 2012; Kteily and Bruneau, 2017). Thus, dehumanizing characteristics describe people that elicit disgust (Sherman and Haidt, 2011). The motivational consequence of disgust is to distance oneself from the target and draw stronger boundaries between the disgusting and dehumanized “others” and oneself (Sherman and Haidt, 2011; Hodson et al., 2014).

Although a prerequisite for understanding dehumanization is to understand what constitutes humanness (Haslam, 2006), humanization does not seem to be the opposite of dehumanization. Christie and Noor (2017) investigated the humanizing and dehumanizing language of politicians describing ethnic groups in Malaysian newspapers by using a coding scheme based on dehumanization characteristics. For example, *lack of culture* was categorized as dehumanizing language, whereas *civility* was categorized as humanizing language. However, Christie and Noor (2017) found that the semantic structure of the humanizing statements was not simply the opposite of dehumanizing statements. Instead, the humanizing statements rather focused on espousing unity and equal treatment between the ethnic groups. Further, characterizing the other ethnic group as caring, cooperative, and contributing to society was also categorized as humanizing statements. These findings indicate that humanizing language is related to the early definitions of humanness: that humanized people are seen as belonging to a community of interconnected individuals whose core tenets include mutual care, respect, and sharing of resources (Kelman, 1973; Opotow, 1990).

Despite these different emphases, dehumanization and humanization imply perceiving others as less and more human, respectively. The difference between the concepts can be understood as a difference in the kind of comparison standard used. Dehumanization results from a perceived violation of a minimal goal of decent human behavior and civility, whereas humanization results from a comparison with a maximal goal of exemplary human behavior (see Brendl and Higgins, 1996; Kessler et al., 2010; Lalot et al., 2018). Thus, dehumanizing is excluding from a community because of a presumed breech of minimal goals, such as following rules set by society, whereas humanizing is perceiving the other as approximating an ideal, such as going out of one’s way to help someone. The extent of dehumanization describes the degree of negative deviation, and the extent of humanization describes how ideal the person or behavior is explains why humanization has no clearly defined endpoint and is more than the absence of dehumanization.

### Kama Muta – An Emotional Route to Viewing Out-Groups as More Human

Outlined above, disgust is an important emotional elicitor of dehumanization. However, to our knowledge, there is no research on the potential effect of positive emotions on viewing out-groups as human. We suggest that kama muta is a positive emotion with consequences for humanization, since it informs people of the opportunity to develop new communal relationships with trustworthy, cooperative people.

Therefore, we expect that kama muta is related to humanization; the characteristics unique to humanness found by the coding scheme of Christie and Noor (2017) are strikingly similar to the characteristics of unity, mutual care, and sharing of resources which are theorized to underlie a CS relation. Kama muta hinges upon the appraisal of suddenly intensified CS between people, which makes the people enacting the communal actions the focus of the emotion eliciting event (Fiske et al., 2019). We therefore expect a strong correlation between kama muta and *individual* humanization of out-group members. We expect this association to reflect both causal directions such that (1) kama muta increases humanization of other individuals and (2) humanization of other individuals increases the level of experienced kama muta. We expect kama muta to increase humanization of out-group members because kama muta is evoked by intense CS, which portrays actions that are considered to be human. Viewing the out-group member as human, which we believe is an important prerequisite for responding emotionally to the out-group member’s actions, is also expected to influence the evocation of kama muta. In other words, we expect that an important prerequisite of feeling kama muta from out-group members’ actions is to see them as people that are able to act in ideal human ways. We will first test for the correlation between kama muta and humanization in the first study and follow up by testing the bi-directionality in the second study.

We expect dehumanization of entire out-groups to be indirectly related to kama muta, through the humanization of individual group members; when kama muta is evoked, out-group members show through their actions that they are part of a close, committed community where people can be trusted to take loyal care of each other. We expect that this altruistic, prosocial, communal commitment of people also licenses the inference that people are *not* amoral, primitive, and irrational and *do not* lack culture. In order for the generalization process from humanization of individuals to reducing the dehumanization of their entire *group* to occur, we expect that further mediating processes will be necessary. Viewing out-group members as human indicates that they are seen as persons who belong in a community. Thus, humanization of individual out-group members should also motivate people to develop CS relationships with out-group members, which reduces dehumanization of the whole out-group. Consistent with this, for instance, perceived friendship potential is an important moderating condition for intergroup contact to produce positive attitudes toward whole out-groups (Pettigrew, 1998).

Furthermore, Haslam (2006) has theorized that construing social relationships in CS terms could further mediate a reduction in dehumanization: people have warm feelings toward their in-group because they trust its members to cooperate and respond to their needs, e.g., by helping them in an emergency. Those who are not part of that in-group are not expected to interact with them in a communal sharing way and are likened to animals. We therefore expect that a feeling thermometer toward the out-group will also mediate the relationship between individual humanization and group dehumanization, as warmth is a key attribute of a CS relation (Schubert et al., 2018). Notably, these chains of mediation imply that the direct effects of kama muta on consequences further downstream are necessarily smaller than those on more proximate consequences, as each link in the chain is only partially influenced by the previous link and partially by extraneous factors. For example, the inference from a person to her group is influenced by group salience, which we increased through our experimental procedures but also by perceived prototypicality of the person, which will vary between perceivers.

## The Current Studies

In the current studies, we investigated the relationship between kama muta elicited through parasocial contact (i.e., watching videos) and viewing out-groups as more human. There has been no such work of which we are aware examining the relationship between positive social emotions and humanization. Therefore, as this is a novel research area, we first conducted a preliminary study, which assessed all previously documented kama muta components (i.e., labels, physiological signs, labels, and CS intensification appraisals) in predicting humanness perceptions of out-groups. We allowed the data-driven procedure to select the best performing kama muta component because there is no consensus in the emotion literature as to which component is central nor how the emotion components affect each other (Scherer, 2019). We also included a range of theoretically derived measures, including potential mediators and moderators, so that we could formally explore in principled ways which of them were best apt for modeling the relationship between kama muta and viewing out-groups as more human (see Supplementary Material).

In our preliminary study, which we describe in detail in the Supplementary Material, we used a formal data-driven approach by employing a machine learning algorithm, called conditional random forest. The output of this analysis provided us with the variables that best predict humanization of individual targets and dehumanization of their entire group. These were kama muta labels, motivation to develop a CS relationship with the out-group protagonist in the video, and warm feelings toward the protagonist’s group. As a last step, we analyzed the relationship between these variables using structural equation modeling to derive a model that is based on both theory and empirical data. Using the conditional random forest method for selecting variables to include in a model is beneficial because it (1) prevents overfitting of models (i.e., mistaking noise in the data for the real signal, see IJzerman et al., 2016) and (2) has much less problems with collinearity than traditional variable selection procedures (IJzerman et al., 2018).

In the preregistered Study 1, we replicated the model derived in the preliminary study using confirmatory analyses on a new sample.

In the preregistered Study 2, we further tested two aspects of the replicated model:

1. We controlled for possibility that the effects of kama muta might simply be accounted for by general positive valence by adding amusement as a comparison condition. Whereas kama muta is associated with the positive experience of fostering close, communal sharing relationships, which should lead to viewing out-groups as more human, amusement leads to a positive, hedonic experience which distracts the viewer from negative thoughts (Oliver and Bartsch, 2010). Thus, if the relational, communal aspect that feeling kama muta signifies is what increases the humanization of individual targets (as we predict), then this effect should be unique to the kama muta videos. If, however, it is simply general positive emotional valence, which increases humanization of the involved parties, then this effect should be solicited by both kama muta and amusing videos.
2. All data and codes are uploaded on OSF: https://osf.io/fmj97/. We report how we determined our sample size, all data exclusions, all manipulations, and all measures. All studies were approved by the internal review board at the Department of Psychology, University of Oslo. Informed consent was obtained from all participants.

We tested the causal directions underlying the previously documented correlation between feeling kama muta and humanizing the protagonist in the video which solicited kama muta; is the protagonist seen as human which then enables the viewer to feel kama muta, or does feeling kama muta enable the viewer to humanize the out-group protagonist, or both?

## Study 1

In the preliminary study, we obtained a model describing the process of how kama muta predicts humanization of individual targets and reduces dehumanization of the entire out-group. Since this model was built based on exploratory analyses, the aim of Study 1 is to use confirmatory analyses to replicate the model obtained in the preliminary study (which is presented in the Supplementary Material). See https://aspredicted.org/fr5w7.pdf for the pre-registration. We will assess the replication of the model in Study 1 through model fit indices.

### Methods

#### Participants

We conducted an *a priori* power analysis based on the RMSEA values of the path-model version of the retained model in the preliminary study (MacCallum et al., 1996). Using an R script by Gnambs (n.d.) with df_model_ = 2, α = 0.05, 1-β (power) = 0.80, and RMSEA at H0 and H1 estimates based on the RMSEA 90% CI values of the retained path model from the preliminary study [0, 0.11], the *a priori* power analysis yielded a sample size of 400^1^.

We recruited *N* = 440 participants from Prolific Academic requesting White heterosexual US American nationals with an approval rate of 90%. The inclusion of only White heterosexual US Americans was in order to ensure that the videos participants saw depicted either ethnic or sexual out-groups. However, some participants categorized themselves as non-heterosexual when responding to demographic questions. Participants were compensated with 1 GBP for their time. As preregistered, participants were excluded from the primary analyses if they did not watch the whole video (*N* = 42), did not watch the video with sound or watched the video with someone (*N* = 24), or were of the same group membership as the protagonist in the video (*N* = 1). We also excluded one participant who was under the age of 18. Of the remaining *N* = 394, 212 indicated that they were male, 33 categorized themselves as non-heterosexual, and all participants categorized themselves as White Americans. Age varied from 18 to 74, *M* = 36.85, *SD* = 13.13.

#### Materials and Procedure

Each participant was presented with one randomly selected moving video from a pool of eight videos (see Supplementary Material for links to videos); The “Thai Medicine” video depicted a little boy being bailed out by a cook for stealing medicine for his sick mother. Years later, the boy now being a doctor, repaid the favor in a much larger scale by paying for the cook’s medical expenses after he had a heart attack. The “Two Orphans” video is about two orphan boys embarking on a journey together to “visit” the mother of one of them. They bond over her grave. The “Thai Altruism” video portrays a day in the life of a man who helps the people around him selflessly. At the end of the video people show their gratitude toward the man. The “Oprah Winfrey” video depicts a reunion between a man and his former music teacher who had helped him get out of poverty. The “Olympics” video shows an athlete getting injured in the middle of a race and continued limping to the finish line, his father then ran on to the field and helped him finish the race. The “Google Search” video depicts a reunion between a Pakistani and Indian man who had not seen each other since the Partition of India. The “Proposal” video shows a man proposing to his boyfriend in front of their friends and family. The “Britain’s Got Talent” video shows a child being nervous and messing up his song which then makes him cry; after being consolidated by his mother and one of the judges, he tries again, sings beautifully and succeeds to the next round of the competition. The videos depicted either Black (i.e., Black American and Black British), Pakistani, Indian, Thai, or gay male protagonists enacting sudden acts of communal sharing. The videos were selected based on that they have been validated in previous studies investigating kama muta (not published and published; Seibt et al., 2017; Schubert et al., 2018; Zickfeld et al., 2019a).

After watching the video, participants were asked to indicate which protagonist they focused on the most during the video (“Later on in the questionnaire you will answer questions about one of the persons you saw in the video. Please select the person you focused on the most during the video clip”). The protagonists were labeled according to name or role (e.g., the father). Scales measured at the group level asked about either Black people, Pakistanis, Indians, Thai people, or gay men, depending on which video participants saw. Then, participants were asked to respond to the dependent and independent variables described below^2^. See Table 1 for example items and descriptive statistics of the measures.

**TABLE 1:** Descriptive statistics and example items for measures used in Study 1.

Scale and example item	N items	Mean (*SD*)	α
Kama muta – physiological signs	3	2.64 (1.60)	0.76
Moist eyes or cried			
Kama muta – appraisals	8	4.02 (1.36)	0.92
I observed an incredible bond			
Kama muta – emotion labels	3	4.42 (1.56)	0.95
It was heartwarming			
Protagonist humanization	3	5.17 (1.03)	0.87
*The protagonist* seemed very human			
Motivation for CS	6	4.23 (1.36)	0.91
*The protagonist* would “give the shirt of their back” for you			
Feeling thermometer in-group^+^	1	76.88 (20.65)	n/a
Please use the scale to indicate how cold or warm you feel toward US Americans			
Feeling thermometer *group presented*^+^	1	70.97 (24.82)	n/a
Please use the scale to indicate how cold or warm you feel toward *the group presented in the video*			
Blatant group dehumanization	9	2.49 (1.07)	0.91
Savage, aggressive			

*Participants were asked to indicate their agreement on scales ranging from 0 to 6, with the exception of the scales marked with ^+^(=0–100). The scales were presented in a fixed order whereas the items within the scales were presented in a random order.*

#### Measures

Participants were asked to indicate their agreement to the following measures using a 7-point scale, ranging from 0 – *Not at all* to 6 – *A lot* with the exception of the feeling thermometer scales which ranged from 0 – *Cold* to 100 – *Warm*.

##### Kama muta

The experience of kama muta was assessed through a measure with three subsections, reflecting physiological signs (three items, “Moist eyes or cried,” “Chills or goosebumps,” and “warm feeling in the chest”), appraisals of communal sharing between the protagonists in the video (eight items, “While watching the video, I observed an incredible bond,” “… a special sense of belonging,” “… an exceptional sense of closeness appear,” “… the emergence of a remarkable feeling of oneness,” “… a unique kind of love spring up,” “… an astonishing sense of someone needing a particular person or being needed by someone,” “… an extraordinary feeling of welcoming or being welcomed,” and “… exceptional care being given to someone”), and the subjective feelings of kama muta (three items, “It was heartwarming,” “I was moved,” and “I was touched”). This kama muta measure was an earlier and shortened version of the measure validated in Zickfeld et al. (2019a). We included the physiological signs and appraisal subscales even though the preliminary study showed that kama muta labels is the best predictor in order to assess that participants felt kama muta.

##### Protagonist humanization

A measure of individual-level humanization was developed for the present study as previous research has focused on dehumanization measures. This face-valid measure of protagonist humanization asked participants to indicate how human they considered the protagonist to be (three items, “[the protagonist] seemed very human,” “[the protagonist]’s actions demonstrate how human the [the protagonist] is,” and “[the protagonist] shows what being human truly is”). These items were directed to the protagonist which participants focused on when watching the video. Thus, the label of the protagonist (e.g., “the father”) was replaced with the protagonist in the items above.

##### Motivation to develop a communal sharing relationship with the protagonist

To assess the extent to which participants were motivated to develop a communal sharing relationship with the protagonist specifically, participants were asked to imagine that they were to get to know the protagonist and rate their agreement to six statements regarding the potential relationship (six items, “[the protagonist] would ‘give the shirt off their back’ for you,” “‘What is mine is yours’ would be true for this relationship,” “You share food with [the protagonist],” “If [the protagonist] needed help, you would cancel plans to give it,” “‘One for all and all for one’ would be true for this relationship,” and “What happens to [the protagonist] is almost as important to you as what happens to you”). These items were taken from Haslam (1994).

##### Feeling thermometers

In order to assess feelings of warmth toward the in-group and out-group, we asked participants to indicate how cold or warm they felt toward US Americans (the in-group), Black people, Pakistanis, Indians, gay men, and Thai people (from Haddock et al., 1993). Because this was a one-item measure, we assessed feelings of warmth toward all groups, and not only toward the out-group which was depicted in the video.

##### Blatant group dehumanization

A measure of blatant dehumanization toward the protagonist’s group (from Kteily and Bruneau, 2017) was given. Participants were asked to rate how well a list of characteristics describe either Black people, Pakistanis, Indians, gay men, or Thai people, based on which video they saw (nine items, “savage, aggressive,” “backward, primitive,” “lacking morals,” “barbaric, cold-hearted,” “refined and cultured,” “rational and logical,” “scientifically/technologically advanced,” “capable of self-control,” and “mature, responsible,” the last five items being reverse scored). This measure is based on the animalistic dehumanization traits described in Bastian et al. (2013), which has been shown to measure blatant dehumanization in studies by Kteily et al. (2015) and Kteily and Bruneau (2017). Blatant dehumanization is the process of explicitly excluding someone from the human category, by using characteristics that are explicitly related to non-human entities when describing them. Ratings of these characteristics correlate moderately with strongly with alignment of out-groups with the “ascent” image of man from quadrupedal human ancestor to modern-day human, indicating that these characteristics underlie blatant dehumanization.

### Results

#### Descriptive Statistics

The descriptive statistics presented in Table 1 show that the videos elicited kama muta in the perceiver, as seen in the above mid-point ratings of kama muta appraisals (*M* = 4.02) and labels (*M* = 4.42). The below mid-point ratings of kama muta physiological signs (*M* = 2.64) indicate that the kama muta experience was not very strong. Protagonist humanization scores were far above the mid-point (*M* = 5.17), and the blatant group dehumanization scores were below the mid-point (*M* = 2.49), indicating the protagonists and their groups were seen as human.

A multivariate analysis of variance (MANOVA) using SPSS 26 was conducted to test for differences between the groups portrayed in the videos. All kama muta components, protagonist humanization, motivation for CS, feeling thermometer score toward the out-group portrayed in the video, and blatant group dehumanization were added as dependent variables. The group portrayed in the video was added as a fixed factor. This factor had five levels, corresponding to Black people (*N* = 158), gay men (*N* = 39), Thai people (*N* = 147), Indians (*N* = 42), and Pakistani (*N* = 8). Both Indians and Pakistanis were portrayed in the same video, so the measure asking participants which protagonist they focused on was used to determine whether they were presented with blatant group dehumanization measures about Pakistanis or Indians.

Across all dependent measures, there was a significant difference between the groups portrayed in the video *F*(28, 1544) = 3.75, *p*<0.001, η*_p_*^2^ = 0.064. Looking at pairwise comparisons (with Bonferroni correction), videos depicting gay men had significantly lower ratings of kama muta labels (*M* = 3.48, 95% CI [3, 3.96]) than videos depicting Black people (*M* = 4.38, 95% CI [4.14, 4.62]), Thai people (*M* = 4.69, 95% CI [4.44, 4.94]), and Indians (*M* = 4.60, 95% CI [4.14, 5.07]). Kama muta physiological signs were also significantly lower among participants who saw a video depicting gay men (*M* = 2.09, 95% CI [1.59, 2.59]) compared to videos depicting Indians (*M* = 3.10, 95% CI [2.61, 3.58]). Gay male protagonists (*M* = 4.63, 95% CI [4.31, 4.95]) were humanized less than Black (*M* = 5.19, 95% CI [5.03, 5.35]) and Thai protagonists (*M* = 5.37, 95% CI [5.20, 5.53]). Participants who saw a video with Thai protagonists were more motivated to develop a CS relationship with the protagonist (*M* = 4.67, 95% CI [4.46, 4.88]) compared to participants who saw videos depicting gay men (*M* = 3.57, 95% CI [3.16, 3.98]) and Black people (*M* = 3.96, 95% CI [3.75, 4.16]). Lastly, Indians received significantly lower blatant group dehumanization scores (*M* = 1.91, 95% CI [1.59, 2.23]) than all of the other groups: Black people (*M* = 2.64, 95% CI [2.48, 2.80]), gay men (*M* = 2.64, 95% CI [2.31, 2.97]), Thai people (*M* = 2.43, 95% CI [2.26, 2.60]), and Pakistanis (*M* = 3.17, 95% CI [2.44, 3.90]).

#### Replicated Model

The model was estimated using latent variable structural equation modeling, specifically the maximum likelihood estimation technique in Mplus version 7 (Muthén and Muthén, 2012). Criteria of model fit were RMSEA <0.08 and upper bound of 90% CI <0.10; CFI >0.95 (Hoyle, 2013) and SRMR <0.08 (Hu and Bentler, 1999). Covariance matrices, measurement models, and information about variable distributions for all models are found in Supplementary Material.

**FIGURE 1:**
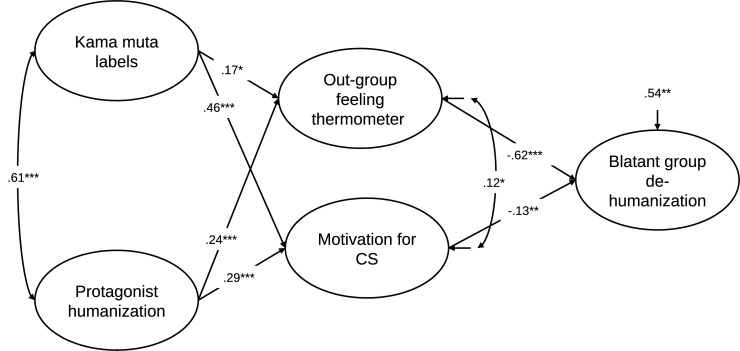
Latent factor model from Study 1 with standardized estimates. *p < 0.05, **p < 0.01, ***p < 0.001.

We estimated the same model as in the preliminary study where kama muta labels and protagonist humanization predicted out-group feeling thermometers (i.e., the group that was depicted in the video) and motivation for CS. Blatant group dehumanization was predicted by out-group feeling thermometers and motivation for CS. The retained structural regression model from the preliminary study was replicated, χ^2^(186) = 396.33, *p* < 0.001, RMSEA (90% CI) = 0.054 (0.046, 0.061), CFI = 0.967, SRMR = 0.045. The standardized parameter estimates are shown in Figure 1. In contrast to the model in the preliminary study, all parameters of the retained model were significant.

We examined the indirect effects of protagonist humanization and kama muta feelings on blatant group dehumanization through motivation for CS or feeling thermometer employing a bootstrap analysis with 10,000 resamples and 95% confidence intervals (Hayes and Scharkow, 2013). This analysis revealed that all indirect paths were significant, meaning that while controlling for protagonist humanization, kama muta feelings predicted a decrease in blatant group dehumanization, and this effect was mediated by both feeling thermometer toward the group presented in the video *B* = -0.062 [-0.123, -0.011] and motivation for CS *B* = -0.036 [-0.077, -0.003], while controlling for the other. Additionally, the effect of protagonist humanization, while controlling for kama muta feelings, predicted a decrease in blatant group dehumanization through feeling thermometer *B* = -0.150 [-0.241, -0.056] and motivation for CS *B* = -0.038 [-0.081, -0.003], while controlling for the other. Comparing the indirect effects (see Preacher and Hayes, 2008) from protagonist humanization, the indirect effect through feeling thermometer was significantly larger than the indirect effect through CS motivation *B* = -0.112 [-0.209, -0.016]. The indirect effects from kama muta feelings were not significantly different *B* = -0.026 [-0.102, 0.041].

### Discussion

The aim of Study 1 was to investigate if the model retained in the preliminary exploratory study would be replicated. We therefore preregistered the model and collected data on a new sample. Our findings show that the model in the preliminary study was replicated in Study 1 as indicated by the model fit indices. Thus, results show that after watching a video that evokes kama muta by depicting communal sharing between out-group members, people view the out-group protagonists as more human. Viewing the individual out-group protagonists as human generalized to ascribing fewer dehumanizing characteristics to their whole group. This effect on blatant out-group dehumanization was mediated by motivation to develop a CS relationship with the protagonist and by having warmer feelings toward the protagonist’s group. The model also shows that kama muta labels also had an indirect effect on blatant group dehumanization through the mediators out-group feeling thermometer and motivation for CS.

However, there was a strong overlap between kama muta labels and protagonist humanization, as seen in the high correlation between these two constructs (*r* = 0.61). We decided to model these as independent variables rather than assuming any directionality by having one predict the other. This is because Study 1 was correlational, making it difficult to assess directionality, and that a bidirectional effect of kama muta on protagonist humanization is plausible. As outlined in the introduction, kama muta can have an effect on protagonist humanization because of the emotion-eliciting event portraying out-groups acting in human ways. Conversely, not viewing the protagonists as human, i.e., as not being capable of acting in human ways, might hinder one from feeling kama muta in response to their actions. Therefore, in Study 2 we will investigate the directionality between kama muta and protagonist humanization.

The comparison of groups depicted in the videos showed that gay men generally evoked less kama muta and were humanized less than the other groups. In addition, participants’ attitudes toward Thai people and Indians were more positive compared to Black people and gay men. Therefore, due to the room for improvement of people’s attitudes toward Black people and gay men we decided to focus on these out-groups in Study 2.

In Study 1, we did not compare the effects in our model with a control condition. The findings in Study 1 do not exclude the possibility that other emotions also have the same effects on viewing out-groups as more human. Therefore, we included videos evoking amusement as a control condition in Study 2. We used amusement because it is also a positive emotion. However, whereas kama muta is related to meaningful positive affect, amusement is a more hedonic experience which distracts the viewer from negative thoughts (Oliver and Bartsch, 2010). Thus, with the inclusion of an amusement condition we were able to test if the effects found in Study 1 were due to positive affect or because of something more specific to kama muta. Lastly, the inclusion of amusement was relevant because, just as is the case with kama muta, a huge amount of online videos and other media video content are produced to evoke amusement (Nelson-Field et al., 2013). This makes amusement and kama muta frequent emotional responses to media video content featuring out-group members, making the understanding of the effects of such emotional responses important for predicting societal processes, particularly social change.

## Study 2

As the model retained in the preliminary study was replicated with confirmatory analyses in Study 1, our aim in this final study is to test two aspects of our model. (1) We test if the effects of kama muta obtained in the preliminary study and Study 1 are due to the specific emotional state of kama muta or whether they can be more generally attributed to its positivity by adding amusement as a comparison condition. (2) Whereas in the preliminary study and in Study 1 we tested for a relation between feelings of kama muta elicited by the video and protagonist humanization, but not its direction, we now aimed to investigate the causal direction of this relationship.

In order to test the directionality of kama muta and protagonist humanization, a pre–post study design with a week delay was employed. In the first time point (i.e., Time 1), participants saw a short segment of a video, which was presented in full at the second time point a week later (i.e., Time 2). At both time points, participants were asked to respond to the protagonist humanization scale after seeing the video. Only a part of the video was presented at Time 1 because we were interested in how human the protagonist was seen *before* the protagonist acted in ways that would either evoke kama muta or amusement. Thus, kama muta and amusement was only measured at Time 2. This design allowed us to test if protagonist humanization measured at Time 1 predicted kama muta scores at Time 2, i.e., if protagonist humanization predicts kama muta. Additionally, by taking the difference score of protagonist humanization between Time 1 and Time 2 and using kama muta scores to predict this difference, we were able to test if kama muta increases protagonist humanization. Furthermore, as blatant group dehumanization was also measured at both Time 1 and Time 2, we were able to test for the directionality of the link between kama muta and blatant group dehumanization.

Thus, the preregistered hypotheses (see https://aspredicted.org/3wc25.pdf for the pre-registration) are as follows:

(H1) Moving videos^3^ will increase protagonist humanization from Time 1 to Time 2, whereas there will be no increase for funny videos.
(H2) Protagonist humanization at Time 1 positively predicts kama muta ratings at Time 2.
(H3) The retained model from the preliminary study and Study 1 will be conceptually replicated with data from Time 2 comparing moving with funny videos.
(H4) Moving videos will decrease blatant group dehumanization, from Time 1 to Time 2, whereas there will be no decrease for funny videos^4^.
(H5) Blatant group dehumanization at Time 1 negatively predicts kama muta ratings at Time 2.

### Methods

#### Participants

We recruited *N* = 350 participants at Time 1 from Academic Prolific requesting White heterosexual US nationals with an approval rate of 90%. The inclusion of only White heterosexual US Americans was in order to ensure that the videos participants saw depicted either ethnic or sexual out-groups. However, some participants categorized themselves as non-heterosexual when responding to demographic questions. Participants were compensated with 1 GBP for their time at Time 1 and were promised an additional 3 GBP after they had completed the study at Time 2. As preregistered, participants were excluded from the primary analyses if they did not complete the second part of the study (*N* = 124); if they were not alone when watching the video (*N* = 9); if they were frequently distracted during the survey (*N* = 2); or if they were of the same group membership as the protagonist in the video (*N* = 8). In addition, we excluded one participant because he had prior knowledge about the protagonist in the video and one participant because he had technical problems. Of the remaining *N* = 221, 122 indicated they were male, all categorized themselves as White/Caucasian and heterosexual. Age varied from 18 to 74, *M* = 34.61, *SD* = 12.19.

#### Materials and Procedure

**FIGURE 2:**
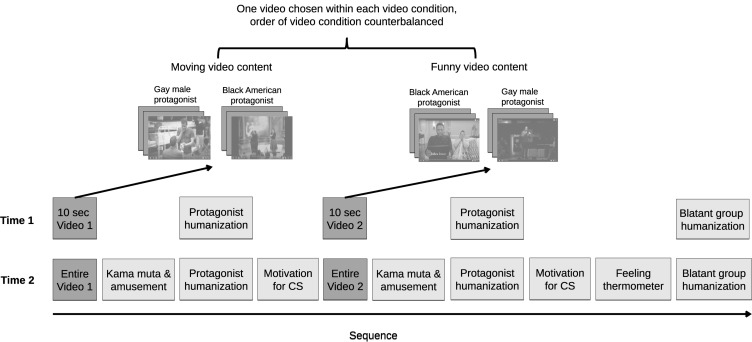
Pictorial representation of the pre and post-test experimental design in Study 2.

A within- and between-subject design over two time points was employed (see Figure 2). Video content was a within-subject factor where participants saw both a funny and a moving video. The between-subject factors were the group membership of the protagonists in the moving and funny videos (i.e., Black American or gay men) and the order in which the videos were presented. Thus, all participants saw both a moving video and a funny video and were presented with both gay male and Black American protagonists, but some participants saw a funny video with a Black American protagonist whereas other participants saw a funny video with a gay male protagonist. There were four sets of videos and participants were only presented with a video from two of the four stimulus sets. These four stimulus sets contained three videos each and portrayed either (1) Black American protagonists evoking kama muta, (2) Black American protagonists evoking amusement, (3) gay male protagonists evoking kama muta, or (4) gay male protagonists evoking amusement. For example, if a participant was presented with a funny video with gay male protagonists, then the second video would be a moving video with a Black American protagonist. The order of all four types of videos was randomized.

At Time 1, participants saw a 10-s segment of the video, selected to not induce kama muta or amusement, in order to give them a frame of reference when responding to the protagonist humanization measure. A week later at Time 2, participants saw the whole video. The videos were presented in the same order at both time points. The group membership of the protagonists was indicated before each video (e.g., “You will now see a video of a Black American father”); this was in order to ensure that participants were aware of the group membership of the protagonists before each video they watched. Furthermore, in order to ensure that responses were based on the video clips alone, and not prior contact or knowledge of the protagonists in the video clips, we asked participants beforehand whether they had heard about the TV series or musician presented in the videos.

The selection of videos for each stimulus set was informed by a pretest. We pretested six moving and six funny videos by measuring amusement, laughter, kama muta labels, and physiological signs (*N* = 72 from Amazon Mechanical Turk, see Supplementary Material) where 10 of these (i.e., five moving and five funny) were selected for the study. Of the videos selected from the pretest that elicited the most kama muta, the “Oprah Winfrey” and “Proposal” videos from Study 1 were used, which depicted Black American and gay male protagonists, respectively. Additionally, the following kama muta videos were also selected based on the results from the pretest: two soldier homecoming videos (intensifying CS by reuniting upon a long separation). The gay male soldier homecoming video was selected based on the pretest, whereas a Black American soldier homecoming video was selected in addition due to the success of the gay male soldier homecoming video in evoking kama muta, but it was not pretested. One wedding video where the Black American groom cries when seeing the Black American bride walk down the aisle (being moved by the intensification of CS that is the formation of marriage) and one video of a colorblind gay man being gifted a pair of glasses that allow him to see color (thus being generously gifted something he truly needs as an act of altruistic communal sharing) were also selected. The videos that were selected based on the results from the pretest that elicited the most amusement were one prank video (which was labeled as having a gay male protagonist), two stand-up videos (with gay male and Black American comedians, respectively), Key and Peele’s “Continental Breakfast” sketch (with Black American protagonist), a scene from Modern Family (with gay male protagonists), and a scene from Black-ish (with Black American protagonists). An additional stand-up video with a gay male comedian (which was not pretested) was selected based on the Black stand-up video’s success in evoking amusement. See Supplementary Material for links to all 12 videos.

#### Measures

Protagonist humanization, blatant group humanization, feeling thermometer, and motivation for CS measures were the same as in Study 1^5^. The group level measures addressed both Black Americans and gay men.

The kama muta measure was slightly modified from Study 1: the physiological sign items from Study 1 were split up to measure one physiological sign at a time (i.e., “moist eyes,” “cried,” “chills,” “goosebumps,” “warm feeling in the chest”). The labels and appraisal components of kama muta were the same as in Study 1. We made composite scores of each kama muta component in addition to a composite score comprising kama muta labels and physiological signs. This is because we wished to include a variable which reflects the feeling and the intensity of kama muta experienced by participants. Amusement was assessed using one item “I was amused”; we also assessed physiological signs (“I laughed”) and appraisal (“I observed something comical”).

Only protagonist humanization and blatant group dehumanization measures were presented at Time 1, whereas at Time 2 all measures were presented (see Figure 2). See Table 2 for descriptive statistics of the measures.

**TABLE 2:** Descriptive statistics for measures used in Study 2.

		Moving		Funny	
			
Scale	Time	*M (SD)*	95% CI	*M* (*SD*)	95% CI
Protagonist humanization	1	4.81 (1.11)	[4.66, 4.96]	4.35 (1.16)	[4.20, 4.50]
	2	5.25 (1.03)	[5.06, 5.44]	3.96 (1.78)	[3.77, 4.15]
Blatant group dehumanization	1	1.65 (1.14)	[1.50, 1.80]	1.61 (1.13)	[1.46, 1.76]
	2	1.65 (1.14)	[1.50, 1.80]	1.60 (1.15)	[1.45, 1.75]
Kama muta	2	2.79 (1.52)	[2.63, 2.96]	0.69 (0.92)	[0.52, 0.86]
Kama muta emotion labels	2	4.17 (1.80)	[3.95, 4.38]	1.08 (1.45)	[0.86, 1.30]
Kama muta appraisals	2	3.92 (1.56)	[3.72, 4.12]	1.42 (1.49)	[1.22, 1.63]
Amusement	2	2.15 (1.94)	[1.89, 2.41]	3.86 (1.99)	[3.60, 4.12]
Laughed	2	1.55 (1.77)	[1.31, 1.80]	3.44 (1.92)	[3.20, 3.68]
Comical	2	1.15 (1.49)	[0.94, 1.37]	4.16 (1.81)	[3.94, 4.38]
Motivation for CS	2	3.84 (1.47)	[3.64, 4.04]	2.47 (1.55)	[2.27, 2.67]
Feeling thermometer^+^	2	73.47 (22.43)	[70.51, 76.43]	73.64 (22.26)	[70.69, 76.60]

*Participants were asked to indicate their agreement on scales ranging from 0 to 6, with the exception of the scales marked with ^+^(=0–100). Kama muta scale was the average of emotion labels and physiological signs (eight items). All scales that were used in Studies 1 and 2 had the same number of items in Study 2.*

### Results

We assessed the internal consistency of the scales measured repeatedly in our analyses by computing multilevel models as recommended by Nezlek (2017): We estimated unconditional three-level hierarchical models in HLM (Raudenbush et al., 2013), with the individual items as measurements at the first level, a variable coding the emotion condition at the second level, and participant at the third level. Estimated item-level reliabilities were 0.70 for protagonist humanization at Time 1, and 0.92 at Time 2; 0.63 for blatant group dehumanization at Time 1, and 0.62 at Time 2; 0.89 for kama muta physiology and labels^6^; and 0.91 for motivation for CS. Nezlek (2017) deemed reliabilities between 0.61 and 0.80 “moderate” and between 0.81 and 1.0 “substantial.” See the Supplementary Material for how missing values were handled.

#### Manipulation Check

We fitted two mixed models using SPSS 24 with kama muta labels and the item amused as dependent variables. Video content (moving vs. funny) was added as a fixed factor, and intercepts were allowed to vary randomly across participants. The main effect of video content on ratings of kama muta labels was significant *F*(1, 220) = 589.39, *p* < 0.001, *B* = 3.09 [2.84, 3.34], and the main effect of video content on amusement ratings was also significant *F*(1, 220) = 114.02, *p* < 0.001, *B* = *-*1.70 [*-*2.02, *-*1.39]. The manipulation was therefore successful (see Table 2).

#### Kama Muta and Protagonist Humanization Path

We tested the hypotheses that kama muta would increase protagonist humanization from Time 1 to Time 2 (H1) and that protagonist humanization at Time 1 would increase evoked kama muta at Time 2 (H2) by fitting mixed models using SPSS 24. In all models, we first added time, group (African American vs. gay male), video content (moving vs. funny), order of video, and their interactions as factors and removed the non-significant factors and interactions from the retained models.

Testing H1, protagonist humanization was added as the dependent variable, and time, group, video content, and the interactions time^∗^video content and video content^∗^group were added as fixed factors. Intercepts were allowed to vary across participants. As predicted, the time^∗^video content interaction was significant, *F*(1, 659) = 34.87, *p* < 0.001, such that protagonist humanization only increased from Time 1 to Time 2 for the moving videos, but not for the funny ones. As seen in the non-overlapping CIs in Table 2, we also found a general main effect such that the initial 10-s video clips from the moving videos had higher protagonist humanization scores than did the 10-s funny video clips. The tests of the remaining effects are reported in Supplementary Table S10.

Exploratorily, we conducted mediation analyses to investigate if it was in fact the elicited emotions that mediated the effect of change in protagonist humanization from Time 1 to Time 2. We tested for mediation using the classical 3-step approach, but fitting mixed models and calculating CIs of the indirect paths using a Monte Carlo procedure (Falk and Biesanz, 2016).

**FIGURE 3:**
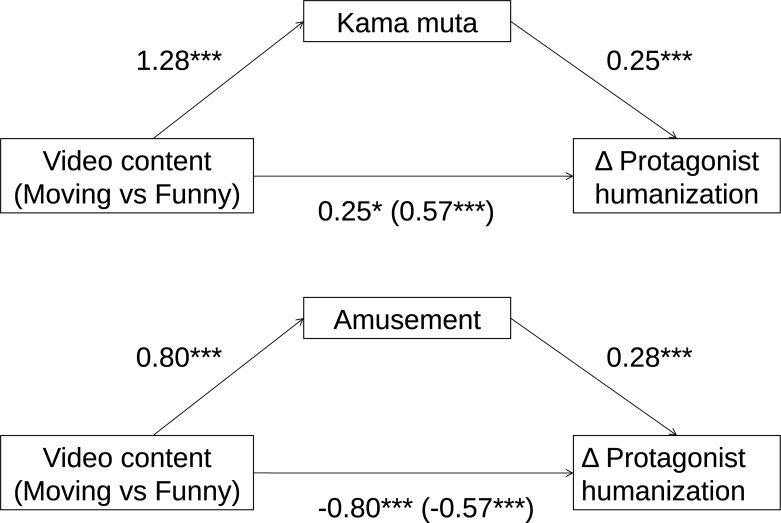
Mediation analyses in Study 2 showing standardized estimates (*z*-values). The coding of video content is reversed in the amusement mediation analyses for ease of interpretation. *p < 0.05, ***p < 0.001.

In the first mediation model, we tested if kama muta (measured with labels and physiological signs) mediated the relationship between video content and change in protagonist humanization from Time 1 to Time 2. We found an indirect effect of kama muta, β = 0.32, *B* = 0.47 [0.25, 0.70], meaning that feeling kama muta in response to the moving video did indeed increase protagonist humanization from Time 1 to Time 2. In the second mediation model, we tested if ratings of amusement mediated the relationship between video content and change in protagonist humanization from Time 1 to Time 2. We did find a positive indirect effect of amusement β = 0.22, *B* = 0.33 [0.21, 0.46], but the direct and total effects of video content on protagonist humanization were negative (see Figure 3). This means that to the degree that participants were *amused* by the funny video, they humanized the protagonist more, as compared to Time 1. However, notice in Table 3 in the means between Time 1 and Time 2 that there was only a small, non-significant (as seen in the overlapping CIs) increase in protagonist humanization for the funny videos when participants had high scores on amusement (+1 SD). Still, *overall*, participants saw the protagonists as less human compared to Time 1 after watching a funny video of them.

**TABLE 3:** Ratings of protagonist humanization in Study 2 while controlling for kama muta and amusement.

Video content	Time	−1 SD	Mean	+1 SD
		**Kama muta as covariate**		

Moving	1	4.42 [4.22, 4.61]	4.81 [4.67, 4.94]	5.19 [5.00, 5.39]
	2	4.70 [4.54, 4.86]	5.25 [5.13, 5.37]	5.80 [5.63, 5.96]
Funny	1	4.18 [3.96, 4.39]	4.35 [4.20, 4.51]	4.53 [4.32, 4.75]
	2	3.25 [2.94, 3.55]	3.96 [3.74, 4.18]	4.67 [4.37, 4.98]

		**Amusement as covariate**		

Moving	1	4.71 [4.50, 4.91]	4.81 [4.66, 4.95]	4.91 [4.70, 5.11]
	2	5.04 [4.85, 5.23]	5.25 [5.12, 5.38]	5.46 [5.27, 5.65]
Funny	1	4.03 [3.82, 4.24]	4.35 [4.21, 4.50]	4.68 [4.47, 4.89]
	2	2.99 [2.71, 3.27]	3.96 [3.76, 4.16]	4.93 [4.65, 5.21]

*Brackets show 95% confidence intervals. The values indicated correspond to the estimated means of blatant group dehumanization at different levels of protagonist humanization ratings at Time 1 and Time 2: –1 SD, Mean and +1 SD of their respective distributions.*

Testing H2, kama muta (labels and physiological signs) was added as the dependent variable, protagonist humanization at Time 1 was added as a fixed covariate, and video content was added as a fixed factor, and their interaction was added as well. Intercepts were allowed to vary across participants. As predicted, the main effect of protagonist humanization at Time 1 was significant, *F*(1, 437.16) = 30.43, *p* < 0.001. In addition, the video content^∗^protagonist humanization at Time 1 interaction was also significant, *F*(1, 301.43) = 21.60, *p* < 0.001, qualifying the main effect. The simple slopes show that the more the protagonist was seen as human before the moving video, the more kama muta participants felt after the moving video, *B* = 0.48 [0.35, 0.62], but not after the funny video, *B* = 0.07 [-0.05, 0.21]. This makes sense, given that the funny videos were not expected to evoke any kama muta, but we had forgotten to preregister that interaction.

Exploratorily, we specified the same model as for H2 but used amusement as the dependent variable. Again, the main effect of protagonist humanization at Time 1 was significant *F*(1, 425.63) = 10.97, *p* = 0.001. So was the video content^∗^protagonist humanization at Time 1 interaction, *F*(1, 321.54) = 5.87, *p* = 0.016, qualifying the main effect. Simple slopes show that the more the protagonist was seen as human before the amusing event, the more amused participants felt after the funny video, *B* = 0.45 [0.24, 0.67], but not after the moving video, *B* = 0.09 [–0.13, 0.32].

#### Structural Equation Modeling

Next, we tested the hypothesis that the retained model from the preliminary study and Study 1 would be conceptually replicated with data from Time 2, comparing moving with funny videos (H3). To do so, we specified a two-condition within-participant path model as suggested by Montoya and Hayes (2017). This procedure includes making a difference score between the two conditions for the mediators and outcome variables in the model (i.e., protagonist humanization, motivation for CS, feeling thermometer, and blatant group dehumanization). We made difference scores by subtracting scores for the funny video from the scores for the moving video, allowing us to investigate the effects of the moving videos relative to the funny videos.

**FIGURE 4:**
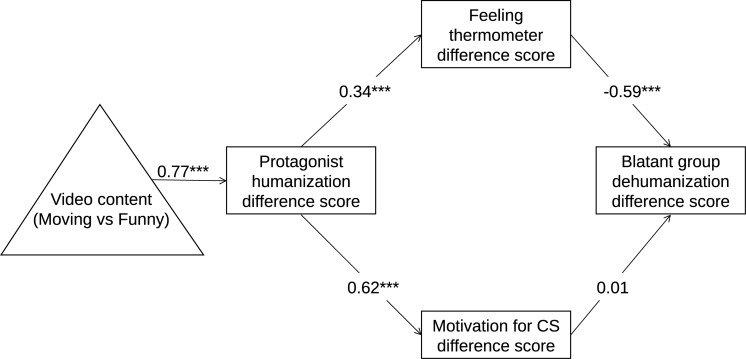
A within-participant serial mediation model from Study 2, with standardized estimates. The direct effect of video content on blatant group humanization (c’) was not significant, *B* = –0.07, *p* = 0.247, whereas a total effect (c) was significant, *B* = –0.19, *p* = 0.003. ***p < 0.001.

We specified a serial mediation path model with the mean difference scores of protagonist humanization, motivation for CS, feeling thermometer toward the out-group, and blatant group dehumanization, all measured at Time 2. We specified a model where the contrast between moving and funny videos predicts protagonist humanization, i.e., that moving videos predict more protagonist humanization than do funny videos^7^. This difference in protagonist humanization, in turn, should result in greater motivation for CS toward the protagonist and warmer feelings toward the protagonist’s group, which in turn should lead to less blatant group dehumanization toward the group (see Figure 4). As seen in Figure 4, the effect of video content is denoted with a triangle, which is common practice when the effect of the variable denoted (i.e., video content) is carried in the difference scores (Montoya and Hayes, 2017).

The model was identified and indicated good model fit, χ^2^(10) = 8.70, *p* = 0.561, RMSEA (90% CI) = 0.00 (0.00, 0.066), CFI = 1.00, SRMR = 0.029. The standardized parameter estimates are shown in Figure 4. Our model shows that the moving video predicted more protagonist humanization, compared to the funny video, and that this difference in protagonist humanization predicted more motivation to develop a CS relationship with the protagonist and warmer feelings toward the protagonist’s group. While controlling for motivation to develop a CS relationship with the protagonist, warmer feelings toward the group after watching the moving video predicted less blatant dehumanization of the group.

We examined the indirect effect of video content on blatant group dehumanization through protagonist humanization, motivation for CS, or feeling thermometer ratings, employing a bootstrap analysis with 10,000 resamples and percentile confidence intervals. This analysis showed a serial mediation from video content to protagonist humanization to warmer feelings of the protagonist’s group to less blatant dehumanization of the said group, *B* = -0.126 [−0.217, −0.049]. The indirect effect through warmer feelings toward the group was significantly larger than the indirect effect through motivation for CS, *B* = –0.129 [−0.266, −0.013]. The indirect effect through motivation for CS was not significant. We also examined a mediation from video content through protagonist humanization to motivation to develop a CS relationship employing the same bootstrap analysis, which showed that this indirect effect was significant, *B* = 0.733 [0.528, 0.960].

#### Kama Muta and Blatant Group Dehumanization Path

Next, we tested the hypotheses that participants exposed to a moving, but not a funny, video would show a decrease in blatant group dehumanization (H4) and that blatant group dehumanization at Time 1 would predict kama muta at Time 2 (H5). We followed the same steps as for H1 and H2.

Testing H4, blatant group dehumanization was the dependent variable, video content and order and the interaction video content^∗^order were fixed factors, as these were the only significant factors that were retained from the initial model. Intercepts were allowed to vary across participants. There was a significant main effect of order *F*(1,661) = 7.81, *p* = 0.005. There was a significant video content^∗^order interaction *F*(1,219) = 3.87, *p* = 0.050, qualifying the main effect. Looking at pairwise comparisons (with Bonferroni correction), when the moving video was presented first, the group depicted in the moving video was blatantly dehumanized more (*M* = 1.72) compared to the group that was presented in the funny video that was presented first (*M* = 1.41), *F*(1,264.27) = 4.86, *p* = 0.028. The blatant dehumanization score for the group depicted in the moving video that was presented second was lower (*M* = 1.58) than for the group that was depicted in the funny video that was presented second (*M* = 1.79), but this difference was not significant, *F*(1,264.27) = 2.39, *p* = 0.123.

Contrary to our predictions, the time^∗^video content interaction on blatant group dehumanization was not significant, *F*(1,651) = 0.008, *p* = 0.93 (see Table 2 for means and SDs). Hence, we did not find evidence that observing a communal sharing interaction, *as compared to* observing an amusing interaction, directly and independently predicts the ratings of blatant group dehumanization from Time 1 to Time 2 to decrease. However, we did find that blatant group dehumanization significantly differed at different levels of protagonist humanization, and this was most prominent for moving videos (see Table 4). Additionally, when controlling for blatant group dehumanization, protagonist humanization still increased from Time 1 to Time 2 for moving videos (see Supplementary Table S13).

**TABLE 4:** Ratings of blatant group dehumanization in Study 2 while controlling for protagonist humanization.

Video content	Time	−1 SD	Mean	+1 SD
		**Protagonist humanization at Time 1 as covariate**		

Moving	1	2.09 [1.86, 2.32]	1.65 [1.51, 1.79]	1.36 [1.17, 1.55]
	2	2.06 [1.83, 2.29]	1.65 [1.51, 1.79]	1.37 [1.28, 1.56]
Funny	1	1.89 [1.71, 2.08]	1.61 [1.47, 1.75]	1.19 [0.97, 1.41]
	2	1.80 [1.61, 1.99]	1.60 [1.45, 1.75]	1.30 [1.07, 1.53]

		**Protagonist humanization at Time 2 as covariate**		

Moving	1	2.49 [2.15, 2.83]	1.65 [1.51, 1.79]	1.29 [1.10, 1.49]
	2	2.46 [2.12, 2.80]	1.65 [1.51, 1.79]	1.31 [1.11, 1.50]
Funny	1	1.76 [1.60, 1.93]	1.61 [1.47, 1.76]	1.25 [1.02, 1.48]
	2	1.79 [1.62, 1.95]	1.60 [1.46, 1.75]	1.16 [0.93, 1.40]

*Brackets show 95% confidence intervals. The values indicated correspond to the estimated means of blatant group dehumanization at different levels of protagonist humanization ratings at Time 1 and Time 2: –1 SD, Mean and +1 SD of their respective distributions.*

Testing H5, kama muta (labels and physiological signs) was added as the dependent variable, blatant group dehumanization at Time 1 was added as a fixed covariate, and video content was added as a fixed factor, and their interaction was added as well. Intercepts were allowed to vary across participants. The main effect of blatant group dehumanization at Time 1 was significant, *F*(1, 351.70) = 16.76, *p* < 0.001, and so was the main effect of video content *F*(1, 242.58) = 223.23, *p* < 0.001. As predicted, the video content^∗^blatant group dehumanization at Time 1 interaction was significant, *F*(1, 254.95) = 9.94, *p* = 0.002, *B* = -0.36 [−0.50, −0.23], qualifying the main effects. Hence, our hypothesis was supported such that the lesser the group was seen as human at Time 1, the lesser participants felt kama muta after the moving video. Also, as predicted, the parameter for the funny video was not significant, *B* = −0.09 [−0.23, 0.05], meaning that blatant group dehumanization did not have an effect on kama muta ratings after the funny video.

### Discussion

The model retained in Study 1 was conceptually replicated in Study 2 in a model where we also compared the effects of moving videos to those of funny videos: Compared to funny videos, moving videos depicting a communal sharing interaction increased protagonist humanization. This difference in protagonist humanization reduced blatant group dehumanization, and this effect was mediated through feeling thermometer scores. In other words, when people see a moving video of specific out-group members acting communally, they see the protagonists as more human. This influences how the entire group of the protagonist is seen; from seeing the group as warm, the whole group is also less dehumanized. We additionally found that prior humanness perceptions of the protagonist and group also affected how much kama muta participants experienced; that is, not only does feeling kama muta make people see others as more human, the more human they see them, the more likely they are to be moved by their communal interactions in the first place.

In contrast to the positive effect of kama muta on humanization of individual out-group members, we found no evidence of a direct, causal effect of video content on blatant dehumanization of the entire out-group. Thus, the impression of the entire out-group, in response to observations of how its members interact, appears to hinge upon the evaluation of its specific, individual members as being human.

Unexpectedly, there was a significant difference in protagonist humanization between the moving video and the funny video at Time 1, i.e., before the emotion-eliciting event. Possibly, some kama muta and amusement could have been elicited during these 10-s video clips, which could have driven the effect. However, as there was an increase in protagonist humanization from Time 1 to Time 2 in the kama muta condition, this does not invalidate the conclusion that watching a moving video leads to increased protagonist humanization.

Also, unexpectedly, we found an order effect of the videos in the blatant group dehumanization responses where the funny video that was presented first had lower blatant dehumanization ratings than the moving video that was presented first. This could be due to our design choice to measure blatant group dehumanization of both groups after participants had seen both moving and funny videos, therefore influencing the blatant group dehumanization scores. The order effect indicates that the funny video attenuated the effect of the moving video on blatant dehumanization, whereas the moving video could have contaminated the blatant dehumanization ratings of the group presented in the funny video. Therefore, even though our design choice produced an unfortunate order effect, we do not see this as invalidating our results as the order effects made the group depicted in the moving video *more* blatantly dehumanized, thus going against our hypothesis and the general pattern of effects that we found. However, future replication work might use a between-subject design to avoid any such order effects.

We did find some unexpected results relating to the funny videos. In the funny video condition, the more participants found the video clip amusing, the more they humanized the protagonist. In addition, the more they saw the protagonist as human to begin with, the more they found the protagonist amusing. This suggests that seeing a character as human enables more emotional reactivity, in the form of feeling both amusement and kama muta. Note, however, that when the funny video was not judged to be amusing (i.e., less than one SD below the mean), protagonist humanization *decreased* from Time 1 to Time 2, whereas even low levels of kama muta increased protagonist humanization at Time 2 (see Table 3).

## General Discussion

In this paper, we have presented novel findings on how a positive emotion, *kama muta*, increases out-group humanization. Previous research has focused on how negative emotions have increased *de*humanization (e.g., Dalsklev and Kunst, 2015), whereas this paper presents a possible way to ameliorate dehumanization, which has been shown to have many negative consequences (e.g., Kteily et al., 2015). The aims of this paper were to investigate the effect of the emotion of kama muta evoked by observing communal interactions between out-group members on viewing them as more human and to investigate which variables mediate the effect of this emotion on the reduction of blatant out-group dehumanization. As predicted, we found that kama muta increases humanness perceptions at both the individual and group levels and that reduction of group-level dehumanization is mediated by feelings of warmth toward the out-group, and humanization of the protagonists depicted in the communal interactions.

In one preliminary study (presented in the Supplementary Material) and two studies, we showed participants’ video clips depicting extraordinary communal sharing interactions (that evoke kama muta) among out-group protagonists. For example, we showed videos of a Pakistani man being reunited with his Indian friend, an African American soldier having a surprise visit to his mother, or a gay man proposing to his boyfriend in the sweetest way. Across all studies, we found that after watching videos that were selected to evoke kama muta, participants consistently reported being *moved*, *touched*, and having a *heartwarming experience*. In addition, ratings of a sudden increase in communal sharing (i.e., kama muta appraisals) were consistently high. Both ratings were also significantly higher for moving videos than for funny videos (as seen in non-overlapping CIs in Table 2). Therefore, we can conclude that we were successful in evoking kama muta in our participants.

Further, our studies also showed that kama muta and protagonist humanization are closely related. Study 1 found a high correlation between the variables so we modeled both as independent variables in our model, not making assumptions regarding how kama muta and protagonist humanization are related. Therefore, in Study 2 we investigated the effect of kama muta on protagonist humanization by measuring protagonist humanization before and after videos evoking kama muta and amusement. As predicted, we found that protagonist humanization increased after watching a kama muta-evoking video depicting out-group protagonists. This effect was stronger than what feeling amusement had on protagonist humanization. How much the viewer perceived the protagonists as human before watching an emotional video also predicted the level of kama muta and amusement in the viewer after watching the video. Viewing the protagonist as human enables people to react emotionally to the video. However, our results do not show whether this prior humanization is due to having more favorable views of the out-groups in the video or if this is an indication of participant’s ability to be transported in the narrative and identify with the protagonists. Future research should look at this.

Study 2 demonstrated that the moving video, relative to the funny video, increased motivation to develop a communal relationship with the protagonist, and this was mediated through protagonist humanization. In other words, when people feel kama muta from watching videos depicting out-group protagonists acting communally, they see these out-group protagonists as more human and therefore as potential partners for committed, close relationships of communal sharing. The kama muta emotional response motivates this psychological process. These results provide support for the proposal that the function of kama muta is to generate increased affective devotion and moral commitment to communal sharing relationships (Fiske et al., 2017). Additionally, Haslam (2006) argued that dehumanization would occur “in social contexts in which relationships are construed in CS terms” (p. 261), i.e., that dehumanization occurs in contexts where one does not see the other person as someone whom one would like to form a CS relationship with. Our findings corroborate Haslam’s (2006) thoughts in showing that when out-group members are regarded as human, they are seen as potential CS relational partners.

Importantly, we also found that humanization of individual targets generalize to reduce dehumanization of their whole group in a model developed through stringent exploratory data analysis in our preliminary study and replicated twice in studies 1 and 2. Kama muta reduces blatant out-group dehumanization through the mediating psychological processes of warm feelings toward the group and motivation to develop a communal sharing relationship with the protagonist. The warmth mediator was the most consistent mediator across the studies. Additionally, viewing the protagonist as human was also an important mediator in the serial mediation model in Study 2.

Our findings provide a social relational explanation for why media that evoke kama muta increase humanization, whereas earlier research by Oliver et al. (2015) and Krämer et al. (2017) provided a cognitive explanation, referring to broadening one’s connection to all of humanity as a result of feeling moral elevation. Kama muta is evoked by viewing people suddenly intensifying their communal sharing relationship, making the protagonists in a moving video central to the appraisal. Therefore, the humanization of the protagonists is an important step in the process of improving the attitudes toward the protagonist’s group. Conversely, Oliver et al. (2015) findings focused on the themes of shared human goodness that the elevating media content illuminate and how this leads to an increased sense of connectedness with humanity and improved attitudes toward many out-groups. However, we have not directly tested if kama muta evoked by one out-group protagonist leads to humanization of another out-group, i.e., whether kama muta leads to an internal change in the perceiver, as Oliver et al. (2015) findings illuminate. Therefore, future studies should test for humanization of out-groups not presented in the kama muta evoking videos.

Having warm feelings toward the protagonist’s group was also an important mediator for reducing blatant dehumanization of the entire out-group. Such “warm” feeling is often conceptualized as indicating lack of emotional prejudice (Haddock et al., 1993), but it could also be seen as indicating communal feelings or empathy toward the group. Indeed, Bruneau et al. (2018) recently found that cold feelings and dehumanization did not predict one another: Whereas feeling thermometer scores are subject to in-group favoritism, some groups see themselves as lower on humanity compared to other (high-status) groups (however, see Rai et al., 2017). Given these findings, it is noteworthy that warm feelings were so closely related to blatant out-group dehumanization in the current studies.

Lastly, some limitations and suggestions for future research are warranted. First, the amusement measure in Study 2 could have been more reliable, i.e., we could have developed an amusement measure as thorough as the KAMMUS measure of kama muta. Future research should therefore focus on this. Second, the order effect of the blatant dehumanization measure in Study 2 indicates that the effect of kama muta is perhaps not very durable: When we presented a funny video between the moving video and the blatant group dehumanization scale, we obtained lower ratings in blatant dehumanization for the group presented in the moving video than when we presented the moving video second and assessed its effect directly afterward. However, this is only an indirect indication about the durability of the effect, and future studies should investigate this question directly. In addition, we expect that the effect of one exposure is not very durable but that the cumulative effect of repeated exposures is. This assumption should also be tested in future research, as it can inform interventions and campaigns aiming at reducing out-group dehumanization and intergroup hostility. Third, moral elevation (Haidt, 2000), which is an emotion often labeled as being moved, was not included as a control emotion in Study 2. This is because there is no validated measure of moral elevation (Pohling and Diessner, 2016; Zickfeld et al., 2019b). Furthermore, there are theoretical and empirical inconsistencies related to what evokes moral elevation. Even though moral elevation is conceptualized to be evoked by moral beauty, studies have found that moral elevation is evoked by films portraying family connectedness, and the importance of love and kindness (Janicke and Oliver, 2017), which is more easily explained by our kama muta framework. Therefore, it was difficult to empirically compare kama muta with moral elevation in Study 2. However, once an authoritative measure of moral elevation will be available, it will be possible in future work to compare the effect of kama muta to that of moral elevation on increasing out-group humanization. Lastly, we presented the protagonist humanization and blatant group dehumanization measures in our studies in a fixed order, which prevents us from controlling for order effects pertaining to these measures.

To conclude, our results suggest that seeing media video content depicting communal interactions between out-group members and feeling kama muta as a response to it will most likely have an effect on viewing these groups as more human. This is precisely what the Norwegian TV series SKAM did for the viewers cited in the beginning of this paper.

## Data Availability Statement

The data that support the findings of this study are openly available in OSF at: https://osf.io/fmj97/.

## Ethics Statement

The studies involving human participants were reviewed and approved by the Internal Review Board at the Department of Psychology, University of Oslo. The participants provided their written informed consent to participate in this study.

## Author Contributions

JB conducted the statistical analyses and wrote the first draft. All authors conceived, designed, conducted the studies, and revised the final manuscript. All authors contributed to the article and approved the submitted version.

## Conflict of Interest

The authors declare that the research was conducted in the absence of any commercial or financial relationships that could be construed as a potential conflict of interest.

## Footnotes

1 As RMSEA is a parsimony-adjusted index of model fit, using the values of a path model with a low number of model degrees of freedom (df_model_ = 2), compared to a latent factor analysis with df_model_ = 186, this power analysis was conservative.

2 Additionally, two measures assessing the protagonist’s and the self’s connectedness with humanity were included for exploratory reasons, which will not be presented in the current paper. Social Dominance Orientation was also measured and findings relating to this measure are presented in the Supplementary Material.

3 In order to distinguish between the dependent and independent variables in the analyses, we will denote the video contents as “moving” and “funny” and the ratings as “kama muta” and “amusing.”

4 A three-item group-level humanization measure developed by the authors was also included in Study 2, and we pre-registered hypotheses similar to H4 and H5 but with this measure. However, the reliabilities for the measure were not acceptable at Time 1 (0.38) nor Time 2 (0.59), leading us to drop these hypotheses.

5 Social Dominance Orientation and Right-Wing Authoritarianism was also measured and findings relating to these measures is presented in Supplementary Material.

6 As pre-registered, we combined the label and physiology items when assessing the bi-directionality of kama muta and (de)humanization (H1, H2, H4, and H5).

7 We did not include kama muta nor amusement measures in the model since the manipulation was successful in that moving videos evoked kama muta, and funny videos amusement.
