# Supplementary material for: Moved by Observing the Love of Others: Kama Muta Evoked Through Media Fosters Humanization of Out-Groups
Source: Front Psychol. 2020 Jun 24;11:1240. doi: 10.3389/fpsyg.2020.01240 (PMC7328370; doi:10.3389/fpsyg.2020.01240)
Supplement: Supplementary file 1 [file Table_1.docx]

Supplemental Materials Table of Contents

[**Preregistrations** 3](#_Toc42626724)

[**Online Materials** 3](#_Toc42626725)

[**Preliminary Study** 5](#_Toc42626726)

[**Supplemental Materials for Study 1** 17](#_Toc42626727)

[**Measurement Model of Replicated Model** 17](#_Toc42626728)

[**SDO Results** 18](#_Toc42626729)

[**Supplemental Materials for Study 2** 21](#_Toc42626730)

[**Pre-Test of Videos** 21](#_Toc42626731)

[**Handling of Missing Values** 23](#_Toc42626732)

[**SDO and RWA Results** 23](#_Toc42626733)

[References 26](#_Toc42626734)

[**Supplemental Tables** 32](#_Toc42626735)

[**Table S1** 32](#_Toc42626736)

[*Descriptive statistics for composite measures in the Preliminary Study* 32](#_Toc42626737)

[**Table S2** 34](#_Toc42626738)

[*Descriptive statistics for scales and items in the Preliminary Study* 34](#_Toc42626739)

[**Table S3** 41](#_Toc42626740)

[*Estimates for the retained measurement model in The Preliminary Study* 41](#_Toc42626741)

[**Table S4** 43](#_Toc42626742)

[*Covariance matrix for retained model in The Preliminary Study* 43](#_Toc42626743)

[**Table S5** 44](#_Toc42626744)

[*Descriptive statistics for scales and items in Study 1* 44](#_Toc42626745)

[**Table S6** 46](#_Toc42626746)

[*Estimates for the retained measurement model in Study 1* 46](#_Toc42626747)

[**Table S7** 48](#_Toc42626748)

[*Covariance matrix for replicated model in Study 1* 48](#_Toc42626749)

[**Table S8** 49](#_Toc42626750)

[*Covariance matrix for model with SDO as control variable in Study 1* 49](#_Toc42626751)

[**Table S9** 50](#_Toc42626752)

[*Results from video pre-test in Study 2* 50](#_Toc42626753)

[**Table S10** 51](#_Toc42626754)

[*Test of H1 in Study 2: Kama muta predicts protagonist humanization* 51](#_Toc42626755)

[**Table S11** 52](#_Toc42626756)

[*Covariance matrix for within-participant model in Study 2* 52](#_Toc42626757)

[**Table S12** 53](#_Toc42626758)

[*Univariate normality of variables in within-participant model in Study 2* 53](#_Toc42626759)

[**Table S13** 54](#_Toc42626760)

[*Ratings of protagonist humanization in Study 2 while controlling for blatant group dehumanization* 54](#_Toc42626761)

[**Table S14** 55](#_Toc42626762)

[*SDO results in Study 2: SDO moderating the effect of kama muta on change in protagonist humanization from Time 1 to Time 2* 55](#_Toc42626763)

[**Table S15** 56](#_Toc42626764)

[*RWA results in Study 2: RWA moderating the effect of kama muta on change in protagonist humanization from Time 1 to Time 2* 56](#_Toc42626765)

[**Supplemental Figures** 57](#_Toc42626766)

[*Figure S1.* Correlation matrix of all composite values in the Preliminary Study. 57](#_Toc42626767)

[*Figure S2.* Permutation importance rankings with blatant group dehumanization as dependent variable from the Preliminary Study. 58](#_Toc42626768)

[*Figure S3.* Permutation importance rankings with protagonist humanization as dependent variable from the Preliminary Study. 59](#_Toc42626769)

[*Figure S4.* Latent factor model from the Preliminary Study with standardized estimates. 60](#_Toc42626770)

[*Figure S5.* Path model from the Preliminary Study with state empathy as third IV, with standardized estimates. 61](#_Toc42626771)

[*Figure S6.* Latent factor model from Study 1 with SDO as a control variable, with standardized estimates. 62](#_Toc42626772)

Supplemental Material

**Preregistrations**

H1 in Study 1 and H1-H5 in Study 2 were preregistered. These can be accessed through these anonymous peer review links:

- Study 1: <https://aspredicted.org/fr5w7.pdf>
- Study 2: <https://aspredicted.org/3wc25.pdf>

**Online Materials**

Data and code are available at: <https://osf.io/fmj97/>

In Studies 1 and 2, we used the following videos:

<https://youtu.be/4C0uhUikeGI>

<https://youtu.be/SAXFKnTIosk>

<https://youtu.be/MdNG33JrfNg>

<https://youtu.be/yN1IJZYBaRw>

<https://youtu.be/fLL-9KA4UMk>

<https://youtu.be/bLVTOP_2GKg>

<https://youtu.be/gHGDN9-oFJE>

<https://youtu.be/W-vGATU1lgU>

In Study 2, we used the following videos:

Amusing videos

*10 second clips (at Time 1)*

<https://www.youtube.com/embed/RXGT9Syj6is?start=42&end=61>

<https://www.youtube.com/embed/uk2Azl628Nw?start=0&end=11>

<https://www.youtube.com/embed/st21dIMaGMs?start=5&end=18>

<https://www.youtube.com/embed/xfKB4MEe9UU?start=38&end=52>

<https://www.youtube.com/embed/dpJkNIYQG2g?rel=0>

<https://www.youtube.com/embed/x81Iv7eSw3A?start=23&end=34>

*Whole videos (at Time 2)*

<https://youtu.be/7XJ1dGGTHnE>

<https://youtu.be/x81Iv7eSw3A>

<https://youtu.be/RXGT9Syj6is>

<https://youtu.be/xfKB4MEe9UU>

<https://youtu.be/uk2Azl628Nw>

<https://youtu.be/st21dIMaGMs>

Kama muta videos

*10 second clips (at Time 1)*

<https://www.youtube.com/embed/sC9ihDuzHKs?rel=0>

<https://www.youtube.com/embed/inkZIfIaF5w?start=0&end=9>

<https://www.youtube.com/embed/FwFJQ0AlAOo?start=41&end=53>

<https://www.youtube.com/embed/W-vGATU1lgU?start=91&end=100>

<https://www.youtube.com/embed/dxyvFHV5OBs?start=7&end=19>

<https://www.youtube.com/embed/hlgQCeJD-UQ?start=0&end=15>

*Whole videos (at Time 2)*

<https://youtu.be/inkZIfIaF5w>

<https://youtu.be/FwFJQ0AlAOo>

<https://youtu.be/hlgQCeJD-UQ>

<https://youtu.be/dxyvFHV5OBs>

<https://youtu.be/W-vGATU1lgU>

<https://youtu.be/MdNG33JrfNg>

**Preliminary Study**

The aim of the preliminary study was to explore how kama muta affects humanness perceptions. We investigate this by following a theoretically guided, data-driven procedure. We start by giving an overview of the rationale for all variables included in the preliminary study.

When feeling kama muta from witnessing intensified communal sharing between out-group members, one is parasocially interacting with these out-group members. Could this positive parasocial interaction with individual out-group members lead to viewing the whole group as more human? Previous research indicates that both direct and parasocial contact with individual out-group members lead to increased humanization of the whole group (Capozza, Trifiletti, Vezzali, & Favara, 2013; Visintin, Voci, Pagotto, & Hewstone, 2017). However, as kama muta theory places large emphasis on the CS relation that is suddenly intensified in the appraisal of the emotion, it could be that humanization occurs first at the individual level. Relatedly, as the measures used in the dehumanization literature focus on *de*humanization, and we were interested in humanization, we developed an individual-level humanization measure for the purposes of our study.

Another question we wanted to illuminate concerns the type of dehumanization that kama muta could affect. Blatant dehumanization has been shown to increase due to negative intergroup contact (i.e., the Boston Marathon Bombings, Kteily et al., 2015), whereas subtle dehumanization is affected by negative emotions such as disgust (Dalsklev & Kunst, 2015). Therefore, in order to address these questions, we included measures of dehumanization that differed in whether they were subtle (Haslam & Bain, 2007; Haslam, Bain, Douge, Lee, & Bastian, 2005; Haslam, Bastian, & Bissett, 2004) or blatant (Kteily & Bruneau, 2017a) and whether they assessed individual- or group-level dehumanization.

We are also interested in the *process* from feeling kama muta through parasocial contact to out-group humanization. As no previous research has investigated the effect of positive social emotions on out-group humanization, we looked to the literature on intergroup contact and media elicited moral elevation, and kama muta theory, for possible mediators.

As kama muta theory asserts that the motivational effect of kama muta is to further intensify the CS relationship that evoked the kama muta feeling, we included a measure assessing motivation to develop a CS relationship with the out-group protagonist in the video. Haslam (2006) has theorized that construing social relationships in CS terms could underlie animalistic dehumanization, where those who are not part of one’s ingroup that interacts in CS terms are likened to animals. Therefore, viewing the protagonist as someone one would like to have a CS relationship with could have downstream effects on out-group humanization. Feeling thermometers toward the out-group were also included, as warmth is a key attribute of a CS relation (Schubert, Zickfeld, Seibt, & Fiske, 2018). Additionally, research on blatant dehumanization has shown that cold feelings (indicating prejudice) and blatant dehumanization are separate constructs (Kteily & Bruneau, 2017b), making it interesting to assess the relationship between perceived out-group warmth and humanization.

Research on intergroup contact and humanization has shown that interacting with out-group members increases one-group representations (i.e., viewing the out-group member and oneself as being part of one group) and state empathy (i.e., having the same feelings as the out-group protagonist) (Capozza, Falvo, Favara, & Trifiletti, 2013; Capozza, Trifiletti, et al., 2013). We included these measures to assess if a similar process as in direct intergroup contact would increase out-group humanization when watching out-group members interacting communally. Research on media-induced elevation showed that elevation increased people’s feeling of being connected with all of humanity (Oliver et al., 2015). We included the connectedness with humanity measure in order to assess if feeling kama muta leads to viewing oneself as a member of a shared human in-group, and if this increases humanization of out-groups.

Lastly, we measured trait empathic concern (Davis, 1983) as a possible moderator. This is because being disposed to feeling compassion towards others in need is related to kama muta (Zickfeld et al., 2017), making it possible that only people high on EC would humanize out-groups as a result of kama muta.

To develop our model, we used a formal data-driven approach by first assessing which (de)humanization measures correlated the most with the variables included in The Preliminary Study and then used these as outcome variables in a machine learning algorithm, called conditional random forest. The output of this analysis provided us with the variables that best predict outgroup humanization, i.e., the best performing sub-scale of kama muta, and mediators. As a last step, we analyzed the relationship between these variables using structural equation modelling to derive a model that is based on both theory and empirical data. Using the conditional random forest method for selecting variables to include in a model is beneficial because it 1) prevents overfitting of models (i.e., mistaking noise in the data for the real signal, see IJzerman, Pollet, Ebersole, & Kun, 2016); and 2) has much less problems with collinearity than traditional variable selection procedures (see IJzerman et al., 2018).

Method

Participants. We recruited *N* = 386 participants to ensure a sample size suitable for structural equation modelling (Barrett (2007) recommends a minimum sample size of 200). Participants were recruited through Amazon Mechanical Turk, requesting only workers from the US and with an approval rate of 95%. Participants were compensated with 2 USD for their time. Participants were excluded from the primary analyses based on the following *a priori* criteria; if they did not watch the whole video (*N* = 29); indicated having the same group membership as the protagonist in the video *(N* = 36*);* did not watch the video with sound (*N* = 2); or did not watch the video alone (*N* = 5). Additionally, we excluded one participant who was under the age of 18 from data analysis, due to our ethics approval. Of the remaining *N* = 313, 156 indicated that they were male, 240 categorized themselves as White/Caucasian, 19 as Asian, 17 as African American, 12 as Mexican or American-Mexican, 11 as other Hispanic, 9 as “other”, 4 as Native American, and 1 as Pacific Islander. Age varied from 19 to 74, *M =* 36.58, *SD =* 11.72.

**Materials and Procedure.** Each participant was presented with one randomly selected moving video from a pool of eight videos (for links to videos see the Online Materials section above). The videos depicted either African American, African British, Asian, or gay protagonists enacting sudden acts of communal sharing, and have been used in previous studies (e.g., Schubert et al., 2018; Seibt, Schubert, Zickfeld, & Fiske, 2017). The videos were the same as in The Preliminary Study of the main manuscript.

After the video, participants were asked to indicate which protagonist they focused on the most during the video (“Later on in the questionnaire you will answer questions about one of the persons you saw in the video. Please select the person you focused on the most during the video clip”). The protagonists were labelled according to name or role (e.g., *the father*). Scales measured at the group level asked about the protagonist’s group (e.g., *the father’s group*). Then participants were asked to respond to the following dependent and independent variables (see also Table S1 for descriptive statistics and example items of each measure and Table S2 for descriptive statistics for each individual item).

**Measures.**

***Kama muta.*** The experience of kama muta was assessed through a measure with five subsections, totaling 55 items, which reflects physical sensations, appraisals of communal sharing between the protagonists in the video, motivation to seek new or renew communal sharing relationships in general (i.e., not related to the protagonist), positive and negative affect, and the subjective feelings of kama muta (i.e., labels). This kama muta measure was an earlier version of the measure validated in Zickfeld (2019).

***Motivation to develop a communal sharing relationship.*** To assess the extent to which participants were motivated to develop a communal sharing relationship with the protagonist specifically, participants were asked to imagine that they were to get to know the protagonist, and rate their agreement to six statements (form Haslam, 1994) regarding the potential relationship.

***Group perception.*** Categorization of the protagonist’s group and the participant’s group as being the same (one-group) or different (two-group) was measured (adapted from Gaertner, Mann, Murrell, & Dovidio, 1989). Additionally, perceptions of having a common in-group identity with the protagonist (adapted from Vezzali et al., 2015) and viewing the protagonist as an individual or group member was measured (developed by the authors).

***Trait and state empathy.*** The state empathy measure was from Capozza, Falvo, et al. (2013). Trait empathic concern was measured with a subscale of the emotional reactivity index (from Davis, 1983).

***Connectedness with humanity.*** This scale asks participants to select from a series of images depicting one small and one big circle moving closer to each other, ending in the small circle completely overlapping the big circle which was labelled “human kind”. Two versions of this measure were used; One where the small circle was labelled “self” and assessed connectedness with humanity, and another where the small circle was labelled “the protagonist” and measured humanization (both adapted from Schubert & Otten, 2002).

***Feeling thermometers.*** In order to assess feelings of warmth toward the in-group and out-group, we asked participants to indicate how cold or warm they felt towards US Americans (the in-group) or the protagonist’s group (the out-group) (both from Haddock, Zanna, & Esses, 1993).

***Protagonist (de)humanization.*** A blatant measure of protagonist humanization was developed by the authors for the present study. This face-valid measure of protagonist humanization asked participants to indicate how human they considered the protagonist to be. The items of this scale were: “[the protagonist] strikes me as very human”, “The way [the protagonist] acts demonstrates what it’s really like to be human”, and “[The protagonist] is an example of the most important parts of being human”.

Additionally, we included Haslam’s dual dehumanization measure (Haslam & Bain, 2007; Haslam et al., 2005, 2004). The ratings of desirability, human nature, and human uniqueness for each of the dual dehumanization characteristics were assessed in order to compute a dual dehumanization composite score. Inspecting the mean scores of human nature ratings for each of the traits (see Table S2), there was no clear indication that some traits were high on HN and others low on HN. All ratings were above the mid-point of the scale (i.e., 3), and the range in the mean ratings was very narrow (min = 3.76, max = 5.05, range = 1.29). Inspecting the mean scores of the uniquely human ratings, however, indicated a difference in whether the traits are seen as uniquely human or not. With the exception of “high-strung”, “reserved”, and “suspicious”, all high UH traits as characterized by earlier research, were above the mid-point of the scale. Similarly, with the exception of “artistic” all low UH traits as characterized by earlier research, were below the mid-point of the scale.

Therefore, we computed two versions of the dual dehumanization scale based on the UH ratings: The first scale measured difference in attribution of high UH traits and low UH traits, where we first made a mean score of the attribution of high UH traits based on our data (i.e., ambitious, analytic, imaginative, passionate, frivolous, irresponsible, broad-minded, humble, conscientious, thorough, disorganized, ignorant, rude, and artistic), and a mean score of the attribution of low UH traits (high-strung, reserved, suspicious, active, curious, friendly, impatient, impulsive, jealous, shy, contented, even-tempered, relaxed, comfortable, simple, timid, uncooperative, and odd). Afterwards, a difference score between the attribution of low UH and high UH traits was computed, where higher values indicate more dehumanization (α = .85). The second measure was within-participants correlations between the attribution of traits and the UH ratings of these traits (see Miranda, Gouveia-Pereira, & Vaes, 2014), where positive values indicate humanization whereas negative values indicate dehumanization (α = .92).

***Blatant group dehumanization.*** Participants were asked to rate how well a list of characteristics related to animalistic dehumanization describe the protagonist’s group (from Kteily & Bruneau, 2017a).

Results

**Variable selection.** We used conditional random forest modelling^[[1]](#footnote-1)^ (Breiman, 2001) to select the most important predictors of dehumanization, which then will be used as the mediators between kama muta and humanization.

As we had included several measures of (de)humanization in our study, we submitted all of our composite measures into a bivariate correlation analysis (see Figure S1 for the correlation matrix) to find which (de)humanization variable was best suited as the outcome variable. The protagonist humanization and blatant group dehumanization variables had the highest correlations with the other variables, making these the best-suited outcome variables. We then submitted these two measures of (de)humanization as outcome variables in two separate random forest analyses, using the R package randomForest and an R script from IJzerman et al. (2018).

The algorithm requests specification of three parameters: “mtry”, “ntree”, and “seed”, which correspond to the number of predictor variables in each tree, the number of trees, and the starting point of the trees, respectively. The square root of the total number of variables is the recommended parameter for mtry, which is 4 in our case, and the recommended number of trees is 1000 (Latinne, Debeir, & Decaestecker, 2001). The permutation importance rankings for blatant group dehumanization (seed = 0214), and protagonist humanization (seed = 0224) can be seen in Figure S1 and Figure S2. Permutation importance is a measure of the difference in prediction accuracy before and after permuting a predictor variable (i.e., altering the variable and its association with the outcome variable, averaged over all trees Strobl, Malley, & Tutz, 2009).

The ranking of variable importance, and not the scores on the permutation importance measure, are the results of interest because the importance scores depend on the characteristics of the bootstrapped data set and the values of the mtry parameters. Therefore, we checked the stability of the models by running them several times with different seeds, and calculated Spearman rank order correlations between the models. We ran each model five times with different seeds, four times with mtry = 4 and ntree = 1000, and once with mtry = 4 and ntree = 10000 (see OSF page for R scripts). The models were very stable for both the model with blatant dehumanization (lowest Spearman rho = .93) and protagonist humanization (lowest Spearman rho = .85).

The main outputs of this analysis are the permutation importance rankings, where these indicate the difference in prediction accuracy before and after permuting a predictor variable (i.e., altering the variable and its association with the outcome variable across all participants, averaged over all trees) while still accounting for the other unpermuted variables. As seen in Figures S1 and S2, the permutation importance rankings show that protagonist humanization predicts blatant group dehumanization, whereas kama muta labels predict protagonist humanization. Additionally, the permutation rankings indicate that feeling thermometer ratings of warmth towards the out-group, motivation for CS, and state empathy are important predictors of both protagonist humanization and blatant group dehumanization.

**Path model results for initial model with state empathy as third IV.** The mean scores of blatant group dehumanization, motivation to develop a communal sharing relationship, kama muta labels (moved, touched, and heartwarming), our own developed protagonist humanization measure, and state empathy, along with feeling thermometer scores for the out-group (which were divided by 10), were added in a path model. The model specifications can be seen in the Figure S5. The model was identified and indicated good model fit *χ2*(3) = 4.15, *p* = .245, RMSEA (90% CI) = .035 (.000, .107), CFI = .997, SRMR = .015.

We examined the indirect effect of protagonist humanization, kama muta labels, and state empathy on blatant group dehumanization through motivation for CS and out-group feeling thermometer employing a bootstrapping analysis with 10,000 resamples and 95% confidence intervals (Hayes & Scharkow, 2013). This analysis showed that all of the indirect effects from the independent variables to the dependent variable was significant, with the exception of the indirect effect from kama muta to blatant group dehumanization through motivation for CS. However, comparing the significant indirect effect with the other indirect effects (Preacher & Hayes, 2008), the analysis showed that the only indirect effects that were significantly different was the indirect effects from kama muta to blatant group dehumanization, B = -.07 [-.15, -.003].

The empathy scale measured the extent to which participants felt the same emotions as the protagonist. Thus, as it was modelled as an independent variable, i.e., at the same level as kama muta labels, we decided not to include the variable in the final model. This was in order to keep the model parsimonious in explaining the effect of kama muta on humanness perceptions. However, future research should indeed investigate the difference between feeling kama muta from viewing intensified CS (i.e., third-person kama muta) and empathy in the form of feeling the same emotions as the protagonists.

**Measurement Model.** The measurement model was specified using latent variable structural equation modelling, specifically, the maximum likelihood estimation technique in M*plus* version 7 (Muthén & Muthén, 2012). Missing values were handled using the full information maximum likelihood method in M*plus*.

The tree items for kama muta labels, four items for protagonist humanization, six items for motivation for CS, and nine items for blatant group dehumanization were specified to load on their corresponding scales. A one indicator factor for feeling thermometer toward out-group was specified by setting the measurement error to .23 (based on test-retest reliability from Haddock, Zanna, & Esses, 1993). The measurement model was identified and indicated poor model fit *χ2*(200) = 862.32, *p* < .001, RMSEA (90% CI) = .103 (.096, .110), CFI = .869, SRMR = .079. However, the relative improvement of the five-factor measurement model from a one-factor measurement model (i.e., loading all items onto one factor) was statically significant *χ2_D_* (9) = 1708.33, *p* < .001, indicating that the scales in the measurement model does not underlie one factor.

Residual covariances were inspected to locate the source of poor model fit of the five-factor model. There were large residual covariances for the indicators of blatant group dehumanization, indicating that the blatant group dehumanization factor was misspecified. The correlations between the items of the blatant group dehumanization scale showed low convergent validity where there were low correlations among reverse scored and non-reverse scored items (*r* from .145 to .612), indicating that there was a method effect in the measurement of blatant dehumanization. Therefore, we re-specified the measurement model to include error covariances between the negatively worded and positively worded (i.e., reverse scored) indicators in the blatant group dehumanization factor in order to account for the method effects of this scale. We used this correlated uniqueness (Marsh & Grayson, 1995) method instead of the more robust correlated-trait correlated-method approach due to identification issues and because our model had only one factor (Lance, Noble, & Scullen, 2002). This re-specified measurement model was identified and indicated good model fit *χ2*(184) = 412.61, *p* < .001, RMSEA (90% CI) = .063 (.055, .071), CFI = .955, SRMR = .059. In addition, the relative improvement from the five factor measurement model was statistically significant *χ2_D_* (16) = 449.71, *p* < .001, indicating that modelling the method effect improved model fit. Therefore, the re-specified measurement model was used in inspecting the relationship between the variables in the structural regression model. See Table S3 for the model results.

**Structural model.** The relationships between the retained variables were investigated using latent variable structural equation modelling, specifically, the maximum likelihood estimation technique in M*plus* version 7 (Muthén & Muthén, 2012). Criteria of model fit (used in all studies) were: RMSEA < .08, and upper bound of 90% CI < .10; CFI > .95 (Hoyle, 2013); and SRMR < .08 (Hu & Bentler, 1999).

We specified the model shown in Figure S4. This model was identified and indicated good model fit, χ2(186) = 413.56, *p* < .001, RMSEA (90% CI) = .063 (.059, .071), CFI = .955, SRMR = .058.

We specified an alternative model where protagonist humanization is specified as a mediator instead of an exogenous independent variable, which also indicated good model fit, χ2(185) = 413.62, *p* < .001, RMSEA (90% CI) = .063 (.055, .071), CFI = .955, SRMR = .058. However, the parameter from protagonist humanization to blatant group dehumanization was not significant in this model (β = -0.01, *p* = .869). Additionally, kama muta labels and protagonist humanization correlated highly (*r* = .82). Due to this, we retained the first model (Figure S4). Comparing the measurement model with the structural model, the decrement in model fit from the retained measurement model was not significant $\chi_{D}^{2}$(2)=.95, *p* > .20, indicating that the model fit did not significantly worsen when free parameters were removed from the structurally just-identified measurement model (Table S4 contains the covariance matrix).

Next, we examined the indirect effect of protagonist humanization and kama muta feelings on blatant group dehumanization through motivation for CS and feeling thermometer employing a bootstrapping analysis with 10,000 resamples and 95% confidence intervals (Hayes & Scharkow, 2013). This analysis showed that while controlling for kama muta feelings, perceived humanness of the protagonist led to warmer feelings toward the group as a whole and controlling for motivation to have a CS relationship with the protagonist, this increase in feeling thermometer ratings of warmth was related to less blatant dehumanization, B = -.28 [-.64, -.01]. The other indirect effects were not significantly different from zero.

Discussion

In the Preliminary Study, we explored the relationship between kama muta and out-group (de)humanization; by first employing the conditional random forest procedure to select the variables that best predict blatant group dehumanization and protagonist humanization, and next using SEM to construct a model with the retained variables. The model obtained indicates that after watching a video that evokes kama muta by depicting communal sharing (as seen by the above mid-point ratings on kama muta appraisals, motivation, labels, and positive affect in Table S1), people view the protagonists as more human, which in turn is associated with viewing the entire out-group as more human. This process is mediated through having warmer feelings toward the out-group. The second independent variable in the model, kama muta labels, did not strongly predict the two mediators. This is likely because protagonist humanization accounted for most of the effect, as suggested by the high correlation between these variables (*r* = .82). We selected this model over the alternative model with protagonist humanization as a mediator because it could be that humanization of the protagonist *prior* to feeling kama muta is a contributing factor to the model. This is somewhat corroborated by the finding that the path from protagonist humanization to blatant group dehumanization was not significant in the alternative model, indicating that protagonist humanization as only predicted by kama muta labels did not predict blatant group dehumanization. Therefore, in Study 2 we will investigate the relationship between kama muta and protagonist humanization further.

Regarding the mediators of the model, notice that variables relating to having increased communal sharing devotion toward protagonists or warm feelings toward the protagonist’s group were selected in the variable selection procedure. This corroborates relational models theorizing on kama muta, which posits that kama muta functionally motivates people to seek out and commit to communal relationships.

**Supplemental Materials for Study 1**

**Measurement Model of Replicated Model**

We first specified a measurement model using latent variable structural equation modelling, specifically, the maximum likelihood estimation technique in M*plus* version 7 (Muthén & Muthén, 2012).

The three items for kama muta labels, four items for protagonist humanization, six items for motivation for CS, and nine items for blatant group dehumanization were specified to load on their corresponding scales, *without* a method effect in the blatant measure, similar to the first measurement model in the Preliminary Study. In addition, a one indicator factor for feeling thermometer toward the out-group presented in the video was also specified, using the same measurement error as in the Preliminary Study (.23). This model was identified and yielded somewhat poor model fit *χ2*(200) = 928.30, *p* < .001, RMSEA (90% CI) = .096 (.090, .102), CFI = .887, SRMR = .063. However, as in the Preliminary Study, the relative improvement of this five-factor measurement model from a one-factor model was statistically significant *χ2_D_* (9) = 2814.40, *p* < .001.

We specified the retained measurement model from the Preliminary Study, i.e., specifying method effects, which was identified and indicated good model fit *χ2*(184) = 396.23, *p* < .001, RMSEA (90% CI) = .054 (.047, .061), CFI = .967, SRMR = .045. The relative improvement from the first five-factor measurement model was statistically significant *χ2_D_* (16) = 532.07, *p* < .001. Therefore, the measurement model of the Preliminary Study was replicated in Study 1, and was used in the structural regression model. See Table S6 for model results.

Comparing the measurement model with the structural model (see main manuscript), as in the Preliminary Study, the decrement in model fit from the measurement model was not significant, $\chi_{D}^{2}$(2)=.013, *p* > .975 indicating that the model fit did not significantly worsen when free parameters were removed from the structurally just-identified measurement model (Table S7 contains the covariance matrix).

**SDO Results**

We included SDO (Ho et al., 2015) as a possible moderator in Study 1. This was because the model retained in the Preliminary Study showed that the mediating variables between kama muta and blatant group dehumanization share variance that is not accounted for by kama muta. Therefore, we tested if this unaccounted variance was explained by SDO in Study 1; it could be that people high on SDO would not change their perceptions toward out-groups due to a kama muta inducing video because of their strongly held beliefs about group-based inequalities. This proposition is supported by longitudinal findings showing that SDO measured at one time-point predicts prejudice toward out-groups measured at another time-point four years later (Kteily et al., 2011). This indicates that SDO is an individual-difference measure that explains the cause of prejudice and is not affected by contextual effects such as feeling kama muta. In addition, people high on SDO have been shown to lack empathy toward subordinate groups (Lucas & Kteily, 2018).

In the retained model in the Preliminary Study, the path allowing the residuals of motivation for CS and feeling thermometer to covary was significant, which indicates that these factors share a variance that is not accounted for by the model. This unaccounted variance could be SDO; it could be that only people low on SDO would increase their motivation to devote communally to the protagonist and to increase their warm feelings toward the protagonist’s group after watching a kama muta inducing video. Whereas people high on SDO would not change their perceptions toward out-groups and out-group individuals due to a kama muta inducing video because of their strongly held beliefs about group-based inequalities. Therefore, in the following study we tested the moderating effect of SDO in the retained model.

**Results.** We investigated the moderating effect of SDO on the paths in the replicated model. As SDO is a continuous latent factor, we employed Maslowsky, Jager, and Hemken's (2015) latent moderated structural equations model method for each path of our model separately. This method includes specifying a null structural model where two latent IVs predict a latent DV without including an interaction term. The second step is to specify a structural model with the latent interaction term. In order to infer moderation, the null model must have adequate model fit, the log-likelihood difference test between the models must be significant, and the interaction term of the alternative model must be significant. None of the paths in our replicated model were moderated by SDO, as none of the interaction effects in the alternative models were significant, therefore rejecting H2.

In an exploratory manner, we investigated an alternative way SDO can influence our model. Therefore, we first specified a measurement model of the SDO scale separately. Re-specification was necessary where we allowed the residuals of the seventh (“We should do what we can to equalize conditions for different groups”) and eighth (“We should work to give all groups an equal chance to succeed”) SDO items to correlate. These items could assess general willingness to take action, which is not accounted for by the social dominance orientation factor. The SDO measurement model yielded adequate model fit *χ2*(19) = 102.95, *p* < .001, RMSEA (90% CI) = .106 (.086, .126), CFI = .954, SRMR = .034. Second, we specified an overall measurement model with the SDO factor included, and this model yielded good model fit *χ2*(374) = 839.02, *p* < .001, RMSEA (90% CI) = .056 (.051, .061), CFI = .945, SRMR = .053.

We then specified a structural regression model where we regressed SDO on all endogenous factors in our replicated model therefore controlling for SDO when estimating the effect of kama muta and humanization on group dehumanization through motivation for CS and feeling thermometer. This model yielded good model fit, *χ2*(376) = 839.44, *p* < .001, RMSEA (90% CI) = .056 (.051, .061), CFI = .946, SRMR = .053. The decrement in model fit from the measurement model was not significant, *χ2_D_* (2) = .42, *p* > .20, meaning that freeing parameters from the measurement model did not worsen model fit. In addition, when controlling for SDO, the correlation between residuals of feeling thermometer and motivation for CS was no longer significant, and when freeing this parameter, model fit did not significantly worsen *χ2_D_* (1) = 2.41, *p* > .05. Making this the retained model, *χ2*(377) = 841.85, *p* < .001, RMSEA (90% CI) = .056 (.051, .061), CFI = .945, SRMR = .053. See Figure S6 for a graphical representation of the SDO model, and Table S8 for covariance matrix. When SDO was controlled for, feeling thermometer scores were not significantly predicted by kama muta feelings (*B* = 0.208, *p* = .096), and blatant group dehumanization was not predicted by CS motivation (*B* = -0.040, *p* = .347).

We examined the indirect effect of protagonist humanization and kama muta feelings on blatant group dehumanization through motivation for CS or feeling thermometer, while controlling for SDO, by employing a bootstrap analysis with 10,000 resamples and 95% confidence intervals. This analysis revealed that while controlling for SDO and kama muta labels, protagonist humanization predicted less blatant group dehumanization through feeling thermometer (*B* = -.120, CI: -0.200, -0.036). All other indirect effects were not significant.

**Discussion.** Contrary to our predictions, the moderation hypothesis was not supported; SDO did not significantly moderate any of the paths in our model. However, adding SDO as a control variable in the model did yield good model fit. It might be that we did not have enough power to detect a moderation effect, though we had enough power to control for the effect of SDO (Marshall, 2007). Therefore, when controlling for SDO, the indirect effect found in the Preliminary Study was also found in Study 1, meaning that irrespective of levels of SDO, viewing protagonists as human increases warmer feelings toward the whole group, which then decreases dehumanization of this group. In addition, when controlling for SDO, it was no longer necessary to allow the residuals between feeling thermometer and CS motivation to correlate, indicating that some of the unaccounted variance in the retained model in the Preliminary Study may have been due to SDO.

**Supplemental Materials for Study 2**

**Pre-Test of Videos**

**Method. *Participants.*** We recruited *N* = 72 participants from Amazon Mechanical Turk, where they were compensated with 3 USD for their time. Thirty-nine indicated they were male, *N* = 51 indicated they were White, *N* = 6 Black, *N* = other Hispanic, *N* = 8 Asian, *N* = 1 Native American, and *N* = 3 other. Fifty-five indicated they were straight, *N* = 12 gay, *N* = 4 bisexual, and *N* = 1 asexual. Age varied from 21 to 64, *M* = 34.38, *SD* = 10.77.

***Materials and procedure.*** In total of 12 videos were pre-tested; six funny videos and six kama muta videos. The emotional content of the videos was a between-subjects factor, meaning that participants watched six videos, where three videos had Black protagonists, and the other three had gay men as protagonists. After each video, participants were asked to rate on 7-point Likert scales the following items: “Moist eyes or cried”, “chills or goosebumps”, “warm feeling in the chest” (constituting kama muta physiology), “I laughed” (amusement physiology item), “it was heartwarming”, “I was moved”, “I was touched” (constituting kama muta labels), “it was funny”, and “it was amusing” (constituting amusement labels).

**Results.** A composite measure of kama muta physiology and labels, and amusement labels, by calculating the mean of the corresponding items, were made for each of the 12 videos. See Table S9 for means and standard deviations of amusement and kama muta ratings. Based on the mean ratings, we decided to use all of the pre-tested kama muta videos, except for the talent show video which was replaced with another soldier homecoming video. Of the amusing videos, we selected all pre-tested videos except for the slapstick video, which was replaced by another stand-up video.

**Links to pre-test videos.** See Online Materials section for links to videos used in Study 2.

Oprah: <https://www.youtube.com/embed/W-vGATU1lgU>

Wedding: <https://www.youtube.com/embed/dxyvFHV5OBs>

Talent show: <https://www.youtube.com/embed/BQkhIV_DGSA>

Soldier homecoming: <https://www.youtube.com/embed/yoV4TWiXI0k>

Colorblind: <https://www.youtube.com/embed/WCcxwieuDH0>

Proposal: <https://www.youtube.com/embed/MdNG33JrfNg>

Stand-up: <https://www.youtube.com/embed/uk2Azl628Nw>

Sketch: <https://www.youtube.com/embed/st21dIMaGMs>

Black-ish: <https://www.youtube.com/embed/RXGT9Syj6is>

Prank: <https://www.youtube.com/embed/7YeVxQ_KMiM>

Slapstick: <https://www.youtube.com/embed/5HmemxyZa8Q>

Modern family: <https://www.youtube.com/embed/xfKB4MEe9UU>

**Handling of Missing Values**

One missing value was found for one item of the blatant group dehumanization scale at Time 1, and this was handled by computing a mean score of blatant group dehumanization at Time 1. There were also missing values for the protagonist humanization items at Time 1, where for seven cases had missing values for all items (meaning that 3.2% of values were missing). Little’s MCAR test of missing values with protagonist humanization items at Time 1 and 2 was significant, χ2(3) = 12.46, *p* = .006, meaning that the missing values of protagonist humanization at Time 1 are not missing completely at random. We imputed the missing values with the expectation maximization algorithm using SPSS 24. In addition, we compared the output of analyses where missing values were imputed with the output of analyses where list-wise deletion of cases with missing values (which is default in SPSS) was implemented. These analyses did not produce significantly different results. Results for testing H1-H2 in the main manuscript are with imputed missing values.

**SDO and RWA Results**

In Study 2 we included both SDO (Ho et al., 2015) and RWA (Zakrisson, 2005) to test if one motivational underpinning of prejudice moderates the effect of kama muta on humanization more than another. RWA predicts prejudice toward out-groups that are seen as violating social norms (for example gays, Crawford, Brandt, Inbar, & Mallinas, 2016) whereas SDO predicts prejudice toward lower status out-groups (Duckitt, 2001; Thomsen et al., 2010). As the videos used in Study 2 portrayed either an ethnic or sexual out-group, it was interesting to investigate if SDO and RWA would moderate the effect of kama muta on humanization differently between the two out-groups. Thus, we tested if SDO and RWA moderates the relationship between emotion condition and change in protagonist humanization from Time 1 to Time 2. This was to see if people high on SDO and RWA are less affected by the kama muta videos in how human they consider the protagonists to be. Our pre-registered hypothesis was: People high on SDO and RWA will have a smaller change in humanization from Time 1 to Time 2 compared to people low on SDO and RWA in the moving condition.

**Results.** To test our pre-registered hypothesis, that people high on SDO and RWA and in the moving condition will have a smaller change in humanization from Time 1 to Time 2 compared to people low on SDO and RWA. We fitted two models with protagonist humanization as dependent variable, time, order, content, and group as fixed factors, SDO (for model 1) and RWA (for model 2) as fixed covariates, and their interactions as fixed factors. We removed all non-significant interactions and factors that were not part of significant interactions from the retained models.

For the model with SDO, we retained a model that included SDO, time, order, content, and group as fixed factors, SDO as fixed covariate, and the interactions time*content, order*SDO, group*SDO, and time*order*content as fixed effects. Intercepts were allowed to vary across participants. Again, this analysis found no evidence that SDO moderates the effect of kama muta on protagonist humanization as the three-way interaction between SDO, time, and content was not significant, *F*(1, 639) = .15, *p* = .696. That is, once people feel kama muta by moving videos, this generally increases their humanization of others for high and low SDO-participants alike. However, the main effect of SDO (*F*(1, 218) = 27.41, *p* < .001) was significant. The interaction between SDO and group (gay versus Black) was significant, *F*(1, 654) = 4.87, *p* = .028, qualifying the main effect. Meaning that participants higher on SDO humanized the African American protagonist less than the gay protagonist. The tests for the remaining effects are reported in S16.

For the model with RWA, the retained model included RWA, time, order, content, and group as fixed factors, and RWA as fixed covariate, and the interactions time*content and group*RWA. Therefore, this analysis revealed that RWA did not moderate the effect of kama muta on protagonist humanization *F*(1, 639) = .69, *p* =.405. Again however, there was a significant interaction effect of group and RWA, *F*(1, 657) = 8.42, *p* = .004, meaning that participants higher on RWA humanized the African American protagonist less than the gay protagonist. The tests for the remaining effects are reported in S17.

Discussion. Contrary to our hypotheses, neither SDO nor RWA moderated the effect of moving videos on humanization. Impressively, this suggests that *irrespectively* of one’s prior propensity to be prejudiced, feeling kama muta from watching outgroup members enact intense situations of communal sharing will increase humanization of them, even though participants high in SDO and RWA humanized African Americans less than they did gays, in line with previous findings that these constructs especially potently predict prejudice towards racial and ethnic outgroups (Duckitt, 2001; Duckitt & Sibley, 2010; Sidanius, Cotterill, Sheehy-Skeffington, Kteily, & Carvacho, 2016; Sidanius & Pratto, 1999).

References

Barrett, P. (2007). Structural equation modelling: Adjudging model fit. *Personality and Individual Differences*, *42*(5), 815–824. https://doi.org/10.1016/j.paid.2006.09.018

Breiman, L. (2001). Random forests. *Machine Learning*, *45*(1), 5–32. https://doi.org/10.1023/A:1010933404324

Capozza, D., Falvo, R., Favara, I., & Trifiletti, E. (2013). The relationship between direct and indirect cross-group friendships and outgroup humanization:emotional and cognitive mediators. *TPM - Testing, Psychometrics, Methodology in Applied Psychology*, *20*(4), 383–397. https://doi.org/10.4473/TPM20.4.6

Capozza, D., Trifiletti, E., Vezzali, L., & Favara, I. (2013). Can intergroup contact improve humanity attributions? *International Journal of Psychology*, *48*(4), 527–541. https://doi.org/10.1080/00207594.2012.688132

Crawford, J. T., Brandt, M. J., Inbar, Y., & Mallinas, S. R. (2016). Right-wing authoritarianism predicts prejudice equally toward “gay men and lesbians” and “homosexuals”. *Journal of Personality and Social Psychology*, *111*(2), e31–e45. https://doi.org/10.1037/pspp0000070

Davis, M. H. (1983). Measuring individual differences in empathy: Evidence for a multidimensional approach. *Journal of Personality and Social Psychology*, *44*(1), 113–126. https://doi.org/10.1037/0022-3514.44.1.113

Duckitt, J. (2001). A dual-process cognitive-motivational theory of ideology and prejudice. *Advances in Experimental Social Psychology*, *33*, 41–113.

Duckitt, J., & Sibley, C. G. (2010). Right-Wing Authoritarianism and Social Dominance Orientation differentially moderate intergroup effects on prejudice. *European Journal of Personality*, *22*(5), 583–601. https://doi.org/10.1002/per.772

Gaertner, S. L., Mann, J., Murrell, A., & Dovidio, J. F. (1989). Reducing intergroup bias: The benefits of recategorization. *Journal of Personality and Social Psychology*, *57*(2), 239–249. https://doi.org/10.1037//0022-3514.57.2.239

Haddock, G., Zanna, M. P., & Esses, V. M. (1993). Assessing the structure of prejudicial attitudes: The case of attitudes toward homosexuals. *Journal of Personality & Social Psychology*, *65*(6), 1105–1118. https://doi.org/10.1037/0022-3514.65.6.1105

Haslam, N. (1994). Categories of social relationship. *Cognition*, *53*(1), 59–90. https://doi.org/10.1016/0010-0277(94)90077-9

Haslam, N. (2006). Dehumanization: An integrative review. *Personality and Social Psychology Review*, *10*(3), 252–264. https://doi.org/10.1207/s15327957pspr1003_4

Haslam, N., & Bain, P. (2007). Humanizing the self: Moderators of the attribution of lesser humanness to others. *Personality and Social Psychology Bulletin*, *33*(1), 57–68. https://doi.org/10.1177/0146167206293191

Haslam, N., Bain, P., Douge, L., Lee, M., & Bastian, B. (2005). More human than you: attributing humanness to self and others. *Journal of Personality and Social Psychology*, *89*(6), 937–950. https://doi.org/10.1037/0022-3514.89.6.937

Haslam, N., Bastian, B., & Bissett, M. (2004). Essentialist beliefs about personality and their implications. *Personality and Social Psychology Bulletin*, *30*(12), 1661–1673. https://doi.org/10.1177/0146167204271182

Hayes, A. F., & Scharkow, M. (2013). The relative trustworthiness of inferential tests of the indirect effect in statistical mediation analysis. *Psychological Science*, *24*(10), 1918–1927. https://doi.org/10.1177/0956797613480187

Hoyle, R. (2013). Modification, presentation, and interpretation. In *Structural equation modeling for social and personality psychology* (pp. 54–74). London, UK: SAGE Publications Ltd. https://doi.org/10.4135/9781446287965

Hu, L. T., & Bentler, P. M. (1999). Cutoff criteria for fit indexes in covariance structure analysis: Conventional criteria versus new alternatives. *Structural Equation Modeling*, *6*(1), 1–55. https://doi.org/10.1080/10705519909540118

IJzerman, H., Lindenberg, S., Dalğar, İ., Weissgerber, S. S. C., Vergara, R. C., Cairo, A. H., … Zickfeld, J. H. (2018). The human penguin project: Climate, social integration, and core body temperature. *Collabra: Psychology*, *4*(1), 1–18. https://doi.org/10.1525/collabra.165

IJzerman, H., Pollet, T., & Ebersole, C. (2016). What predicts stroop performance? A conditional random forest approach. *SSRN Electronic Journal*. https://doi.org/10.2139/ssrn.2805205

Kteily, N., & Bruneau, E. (2017a). Backlash: The politics and real-world consequences of minority group dehumanization. *Personality and Social Psychology Bulletin*, *43*(1), 87–104. https://doi.org/10.1177/0146167216675334

Kteily, N., & Bruneau, E. (2017b). Darker demons of our nature: The need to (re)focus attention on blatant forms of dehumanization. *Current Directions in Psychological Science*, *26*(6), 487–494. https://doi.org/10.1177/0963721417708230

Kteily, N., Sidanius, J., & Levin, S. (2011). Social dominance orientation: Cause or “mere effect”?. Evidence for SDO as a causal predictor of prejudice and discrimination against ethnic and racial outgroups. *Journal of Experimental Social Psychology*, *47*(1), 208–214. https://doi.org/10.1016/j.jesp.2010.09.009

Lance, C. E., Noble, C. L., & Scullen, S. E. (2002). A critique of the correlated trait-correlated method and correlated uniqueness models for multitrait-multimethod data. *Psychological Methods*, *7*(2), 228–244. https://doi.org/10.1037//1082-989X.7.2.228

Latinne, P., Debeir, O., & Decaestecker, C. (2001). Limiting the number of trees in random forests. In J. Kittler & F. Roli (Eds.), *Multiple Classifier Systems. MCI 2001. Lecture Notes in Computer Science, Vol. 2096.* (pp. 178–187). Berlin, Heidelberg: Springer. Retrieved from https://link.springer.com/content/pdf/10.1007%2F3-540-48219-9.pdf

Lucas, B. J., & Kteily, N. S. (2018). (Anti-)egalitarianism differentially predicts empathy for members of advantaged versus disadvantaged groups. *Journal of Personality and Social Psychology*, *114*(5), 665–692. https://doi.org/10.1037/pspa0000112

Marsh, H. W., & Grayson, D. (1995). Latent variable models of multitrait-multimethod data. In R. H. Hoyle (Ed.), *Structural equation modeling: Concepts, issues, and applications*  (pp. 177–198). Thousand Oaks, CA: Sage Publications. Retrieved from http://psycnet.apa.org/record/1995-97753-010

Marshall, S. W. (2007). Power for tests of interaction: Effect of raising the Type I error rate. *Epidemiologic Perspectives and Innovations*, *4*, 1–7. https://doi.org/10.1186/1742-5573-4-4

Maslowsky, J., Jager, J., & Hemken, D. (2015). Estimating and interpreting latent variable interactions. *International Journal of Behavioral Development*, *39*, 87–96. https://doi.org/10.1177/0165025414552301

Miranda, M., Gouveia-Pereira, M., & Vaes, J. (2014). When in Rome.. Identification and acculturation strategies among minority members moderate the dehumanisation of the majority outgroup. *European Journal of Social Psychology*, *44*(4), 327–336. https://doi.org/10.1002/ejsp.2025

Muthén, L. K., & Muthén, B. O. (2012). Mplus (Version 7). Los Angeles, CA: Muthén & Muthén.

Preacher, K. J., & Hayes, A. F. (2008). Asymptotic and resampling strategies for assessing and comparing indirect effects in multiple mediator models. *Behavior Research Methods*, *40*(3), 879–891. https://doi.org/10.3758/BRM.40.3.879

Schubert, T. W., & Otten, S. (2002). Overlap of self, ingroup, and outgroup: Pictorial measures of self-categorization. *Self and Identity*, *1*(4), 353–376. https://doi.org/10.1080/152988602760328012

Schubert, T. W., Zickfeld, J. H., Seibt, B., & Fiske, A. P. (2018). Moment-to-moment changes in feeling moved match changes in closeness, tears, goosebumps, and warmth: time series analyses. *Cognition and Emotion*, *32*(1), 174–184. https://doi.org/10.1080/02699931.2016.1268998

Seibt, B., Schubert, T. W., Zickfeld, J. H., & Fiske, A. P. (2017). Interpersonal closeness and morality predict feelings of being moved. *Emotion*, *17*(3), 389–394. https://doi.org/10.1037/emo0000271

Sidanius, J., Cotterill, S., Sheehy-Skeffington, J., Kteily, N., & Carvacho, H. (2016). Social Dominance Theory: Explorations in the psychology of oppression. In C. G. Sibley & F. K. Barlow (Eds.), *The Cambridge handbook of the psychology of prejudice* (pp. 149–187). Cambridge: Cambridge University Press. https://doi.org/10.1017/9781316161579.008

Sidanius, J., & Pratto, F. (1999). *Social dominance: An intergroup theory of social hierarchy and oppression*. Cambridge: Cambridge University Press.

Strobl, C., Malley, J., & Tutz, G. (2009a). An introduction to recursive partitioning: Rationale, application, and characteristics of classification and regression trees, bagging, and random forests. *Psychological Methods*, *14*(4), 323–348. https://doi.org/10.1037/a0016973

Strobl, C., Malley, J., & Tutz, G. (2009b). Supplementary materials. https://doi.org/10.1037/a0016973

Thomsen, L., Green, E. G. T., Ho, A. K., Levin, S., van Laar, C., Sinclair, S., & Sidanius, J. (2010). Wolves in sheep’s clothing: Sdo asymmetrically predicts perceived ethnic victimization among white and latino students across three years. *Personality and Social Psychology Bulletin*, *36*(2), 225–238. https://doi.org/10.1177/0146167209348617

Vezzali, L., Stathi, S., Crisp, R. J., Giovannini, D., Capozza, D., & Gaertner, S. L. (2015). Imagined intergroup contact and common ingroup identity an integrative approach. *Social Psychology*, *46*(5), 265–276. https://doi.org/10.1027/1864-9335/a000242

Visintin, E. P., Voci, A., Pagotto, L., & Hewstone, M. (2017). Direct, extended, and mass-mediated contact with immigrants in Italy: their associations with emotions, prejudice, and humanity perceptions. *Journal of Applied Social Psychology*, *47*(4), 175–194. https://doi.org/10.1111/jasp.12423

Zakrisson, I. (2005). Construction of a short version of the Right-Wing Authoritarianism ( RWA ) scale, *39*, 863–872. https://doi.org/10.1016/j.paid.2005.02.026

Zickfeld, J. H., Schubert, T. W., Seibt, B., Blomster, J. K., Arriaga, P., Basabe, N., … Fiske, A. P. (2019). Kama muta: Conceptualizing and measuring the experience often labelled being moved across 19 nations and 15 languages. *Emotion*, *19*(3), 402–424. https://doi.org/10.1037/emo0000450

**Supplemental Tables**

**Table S1**

*Descriptive statistics for composite measures in the Preliminary Study*

| Scale & Example item | N items | Mean (SD) | α |
| --- | --- | --- | --- |
| Kama muta – physical sensations  Moist eyes | 19 | 1.92 (1.37) | .92 |
| Kama muta – appraisals  I observed an incredible bond | 15 | 3.69 (1.44) | .91 |
| Kama muta – motivation  I felt like telling someone how much I care for them | 9 | 3.00 (1.77) | .93 |
| Kama muta – positive affect  I had positive feelings | 1 | 4.74 (1.51) | n/a |
| Kama muta – negative affect  I had negative feelings | 1 | 1.11 (1.64) | n/a |
| Kama muta – emotion labels  It was heartwarming | 3 | 4.41 (1.64) | .93 |
| Two group  Right after the video clip it felt like you and *the protagonist* were members of **two** separate groups | 1 | 1.49 (1.89) | n/a |
| One group  Right after the video clip it felt like you and *the protagonist* were members of **one** same group | 1 | 3.74 (2.04) | n/a |
| Protagonist humanization  *The protagonist* strikes me as very human | 3 | 4.92 (1.17) | .80 |
| Common in-group identity^*^  My identity, in a sense, also includes *the protagonist’s* identity | 1 | 4.34 (1.86) | n/a |
| Individuation^*^  During the video clip the protagonist was: mostly an individual – mostly a member of a group | 1 | 3.25 (2.20) | n/a |
| Empathy  Feel in tune with him/her | 4 | 4.10 (1.55) | .92 |
| Motivation for CS  *The protagonist* would ‘give the shirt of their back’ for you | 6 | 4.13 (1.48) | .93 |
| Connectedness of self to humanity^*^  Please choose a picture that best represents the current closeness of yourself and humankind | 1 | 3.64 (1.83) | n/a |
| Connectedness of *the protagonist* to humanity^*^  … current closeness of *the protagonist* and humankind | 1 | 5.06 (1.51) | n/a |
| Feeling thermometer in-group^+^  Please use the scale to indicate how cold or warm you feel towards US Americans | 1 | 76.58 (21.11) | n/a |
| Haslam’s dehumanization – difference score  Ambitious | 32 | -0.36 (.56) | .85 |
| Haslam’s dehumanization – within participant correlation  Analytic | 64 | .04  (.25) | .92 |
| Feeling thermometer *the protagonist’s* group^+^  Please use the scale to indicate how cold or warm you feel towards *the protagonist’s* group | 1 | 79.61 (22.88) | n/a |
| Blatant group dehumanization  Savage, aggressive | 9 | 2.38 (1.11) | .89 |
| Empathic concern^x^  I often have tender, concerned feelings for people less fortunate than me | 7 | 4.11  (.85) | .90 |

*Note. The protagonist* was replaced with the name of the protagonist participants focused on the most during the video. Participants were asked to indicate their agreement on scales ranging from 0 to 6, with the exception of the scales marked with ^*^ (= 1-7), ^+^ (= 0-100), and ^x^ (1-5). There were 10 items for kama muta emotion labels but after recommendations of Zickfeld et al. (2019) three items were used. See Supplemental Materials for further information about the Haslam dehumanization measures. The scales were presented in a fixed order whereas the items within the scales were presented in a random order. Means and standard deviations are calculated after imputing missing values.

**Table S2**

*Descriptive statistics for scales and items in the Preliminary Study*

| Scale | Kurtosis | Skew | Missing | Mean | SD |
| --- | --- | --- | --- | --- | --- |
| Kama muta – physical sensations |  |  |  |  |  |
| Moist eyes | -1.44 | 0.37 |  | 2.34 | 2.30 |
| Tears | -0.73 | 0.89 |  | 1.64 | 2.09 |
| Goosebumps or hair standing up | -0.52 | 0.97 |  | 1.54 | 1.98 |
| Chills or shivers | -0.34 | 1.01 |  | 1.38 | 1.80 |
| A warm feeling in the center of the chest | -1.30 | -0.10 |  | 2.89 | 2.06 |
| Some feeling in the center of the chest | -1.33 | 0.32 | 0.3% | 2.23 | 2.06 |
| Choked up | -1.39 | 0.35 |  | 2.27 | 2.17 |
| A lump in the throat | -1.25 | 0.45 |  | 2.14 | 2.13 |
| Difficulty speaking | 3.09 | 2.00 |  | 0.68 | 1.31 |
| I put one or both hands to my chest | 2.92 | 2.05 |  | 0.74 | 1.56 |
| I took a deep breath or held my breath | -0.49 | 0.90 |  | 1.45 | 1.78 |
| I said something like “awww” | -1.45 | 0.32 |  | 2.31 | 2.25 |
| Sneezed or felt like sneezing^d^ | 22.68 | 4.57 |  | 0.21 | 0.76 |
| Headache^d^ | 22.24 | 4.57 |  | 0.23 | 0.85 |
| Laughed or giggled^d^ | 2.23 | 1.84 |  | 0.85 | 1.59 |
| Sick to my stomach^d^ | 11.53 | 3.38 | 0.3% | 0.37 | 1.03 |
| Dizzy^d^ | 23.82 | 4.53 |  | 0.23 | 0.76 |
| Buoyant or light | -1.29 | 0.05 |  | 2.58 | 2.00 |
| Refreshed, energized, or exhilarated | -1.25 | -0.04 |  | 2.74 | 1.98 |
| Kama muta – appraisals |  |  |  |  |  |
| I observed an incredible bond | 0.24 | -1.05 |  | 4.31 | 1.81 |
| … a special sense of belonging | -1.21 | -0.27 |  | 3.19 | 2.07 |
| … an exceptional sense of closeness appear | -0.53 | -0.75 |  | 3.89 | 1.92 |
| … the emergence of a remarkable feeling of oneness | -1.21 | -0.25 | 0.3% | 3.12 | 2.07 |
| … a unique kind of love spring up | -0.80 | -0.58 |  | 3.67 | 1.96 |
| … a phenomenal feeling of being appreciated | -1.11 | -0.42 |  | 3.45 | 2.08 |
| … an astonishing sense of being needed | -1.33 | 0.05 |  | 2.75 | 2.09 |
| … an extraordinary feeling of being welcomed | -1.26 | -0.30 |  | 3.22 | 2.12 |
| … exceptional care being given to someone | 0.68 | -1.17 |  | 4.47 | 1.66 |
| … a great kindness | 1.63 | -1.48 |  | 4.79 | 1.54 |
| … something extremely upsetting^d^ | 1.41 | 1.59 |  | 1.03 | 1.69 |
| … an unusual feeling of anxiety^d^ | 3.52 | 2.04 |  | 0.69 | 1.29 |
| … a feeling being exceptionally horrified^d^ | 7.45 | 2.80 |  | 0.53 | 1.27 |
| … an interaction that was really confusing^d^ | 5.74 | 2.53 |  | 0.60 | 1.36 |
| … an incident that was terribly boring^d^ | 6.97 | 2.69 |  | 0.55 | 1.25 |
| Kama muta – motivations |  |  |  |  |  |
| I felt like telling someone how much I care about them | -1.42 | -0.16 |  | 3.01 | 2.21 |
| I wanted to hug someone | -1.46 | -0.13 |  | 3.06 | 2.24 |
| I wanted to do something extra-nice for someone | -1.08 | -0.42 |  | 3.40 | 2.03 |
| I felt especially friendly | -1.14 | -0.35 |  | 3.26 | 2.03 |
| I felt more strongly committed to a relationship | -1.40 | 0.13 |  | 2.51 | 2.11 |
| I felt like going away to be by myself^d^ | 2.88 | 1.96 |  | 0.81 | 1.53 |
| I felt like yelling at someone^d^ | 15.70 | 3.90 |  | 0.28 | 0.95 |
| I was eager to tell my friends or family about the experience | -1.30 | 0.29 |  | 2.37 | 2.12 |
| I like to have the experience together with others | -1.08 | -0.36 |  | 3.17 | 2.00 |
| Kama muta – affect |  |  |  |  |  |
| I had positive feelings | 1.40 | -1.37 |  | 4.74 | 1.51 |
| I had negative feelings | 1.10 | 1.46 |  | 1.11 | 1.64 |
| Kama muta – emotion labels |  |  |  |  |  |
| It was heartwarming | 1.06 | -1.29 |  | 4.62 | 1.62 |
| I was moved | 0.04 | -1.00 |  | 4.28 | 1.81 |
| I was touched | 0.15 | -1.07 |  | 4.34 | 1.82 |
| It was a nostalgic moment | -1.42 | 0.21 |  | 2.54 | 2.21 |
| It was a poignant experience | -1.09 | -0.37 |  | 3.40 | 2.07 |
| I felt a part of something larger than myself | -1.44 | -0.14 |  | 2.96 | 2.16 |
| I felt in love | -1.13 | 0.60 |  | 2.04 | 2.18 |
| I felt sad^d^ | -0.49 | 0.87 |  | 1.76 | 1.96 |
| I felt great respect^d^ | -0.08 | -1.00 |  | 4.20 | 1.90 |
| I was proud^d^ | -1.33 | -0.20 |  | 3.08 | 2.14 |
| Right after the video clip it felt like you and *the protagonist* were members of **two** separate groups | 0.08 | 1.11 |  | 1.49 | 1.89 |
| Right after the video clip it felt like you and *the protagonist* were members of **one** same group | -0.90 | -0.56 |  | 3.74 | 2.04 |
| Protagonist humanization |  |  |  |  |  |
| *The protagonist* strikes me as very human | 6.29 | -2.36 |  | 5.33 | 1.13 |
| The way *the protagonist* acts demonstrates what it’s really like to be human | 1.65 | -1.40 |  | 4.79 | 1.47 |
| *The protagonist* is an example of the most important parts of being human | 0.71 | -1.14 |  | 4.65 | 1.51 |
| Common in-group identity^*^ |  |  |  |  |  |
| My identity, in a sense, also includes *the protagonist’s* identity | -0.85 | -0.39 |  | 4.34 | 1.86 |
| Individuation^*^ |  |  |  |  |  |
| During the video clip the protagonist was: (1 mostly an individual – 7 mostly a member of a group) | -1.31 | 0.43 |  | 3.25 | 2.20 |
| Empathy |  |  |  |  |  |
| Feel in tune with him/her |  |  |  |  |  |
| Feel you share his/her emotions | -0.27 | -0.75 |  | 3.83 | 1.78 |
| Understand his/her feelings | -0.12 | -0.82 |  | 4.03 | 1.76 |
| Share his/her sorrows | 0.99 | -1.23 |  | 4.56 | 1.56 |
| Motivation to develop a CS relationship | -0.13 | -0.83 |  | 3.96 | 1.77 |
| *The protagonist* would ‘give the shirt of their back’ for you |  |  |  |  |  |
| ‘What is mine is yours’ is true for this relationship | -0.08 | -0.90 |  | 4.16 | 1.77 |
| You share food with this person | -0.40 | -0.74 |  | 3.97 | 1.81 |
| If this person needed help, you could cancel plans to give it | 0.79 | -1.22 |  | 4.59 | 1.65 |
| ‘One for all and all for one’ is true for this relationship | 0.57 | -1.06 |  | 4.38 | 1.60 |
| What happens to this person is almost as important to you as what happens to you | -0.10 | -0.80 |  | 4.06 | 1.73 |
| Connectedness of self to humanity^*^ | -0.68 | -0.50 |  | 3.64 | 1.83 |
| Connectedness of *the protagonist* to humanity^*^ | -0.29 | -0.52 | 0.3% | 5.06 | 1.51 |
| Feeling thermometer US Americans (in-group)^+^ | 1.59 | -1.16 |  | 76.58 | 21.11 |
| Dual dehumanization – high UH and HN |  |  |  |  |  |
| Ambitious | -0.47 | -0.75 |  | 3.99 | 1.87 |
| Analytic | -0.80 | 0.10 | 0.3% | 2.53 | 1.74 |
| Imaginative | -0.22 | -0.61 |  | 3.76 | 1.71 |
| Passionate | 2.30 | -1.57 |  | 4.85 | 1.44 |
| High-strung | 1.57 | 1.53 |  | 0.93 | 1.38 |
| Frivolous | 3.54 | 1.97 |  | 0.73 | 1.22 |
| Irresponsible | 8.27 | 2.74 |  | 0.53 | 1.09 |
| Reserved | -1.04 | 0.13 |  | 2.43 | 1.78 |
| Dual dehumanization – high UH and low HN |  |  |  |  |  |
| Broad-minded | -0.42 | -0.66 |  | 3.81 | 1.78 |
| Humble | 1.04 | -1.21 |  | 4.61 | 1.50 |
| Conscientious | -0.06 | -0.91 |  | 4.17 | 1.78 |
| Thorough | -0.60 | -0.50 |  | 3.59 | 1.79 |
| Disorganised | 6.04 | 2.40 |  | 0.57 | 1.06 |
| Ignorant | 7.21 | 2.66 |  | 0.50 | 1.07 |
| Rude | 16.88 | 3.82 |  | 0.33 | 0.87 |
| Suspicious | 6.59 | 2.51 | 0.3% | 0.57 | 1.13 |
| Dual dehumanization – low UH and high HN |  |  |  |  |  |
| Active | 0.41 | -1.04 | 0.3% | 4.23 | 1.69 |
| Curious | -0.54 | -0.48 |  | 3.46 | 1.77 |
| Friendly | 2.85 | -1.59 |  | 4.88 | 1.31 |
| Artistic | -1.10 | -0.15 |  | 3.11 | 1.99 |
| Impatient | 2.96 | 1.86 |  | 0.70 | 1.15 |
| Impulsive | -0.95 | 0.65 |  | 1.83 | 1.90 |
| Jealous | 6.80 | 2.55 |  | 0.48 | 1.00 |
| Shy | -0.61 | 0.69 |  | 1.73 | 1.73 |
| Dual dehumanization – low UH and low HN |  |  |  |  |  |
| Contented | -0.77 | -0.45 |  | 3.52 | 1.88 |
| Even-tempered | -0.01 | -0.84 | 0.3% | 4.07 | 1.70 |
| Relaxed | -0.83 | -0.40 | 0.3% | 3.39 | 1.80 |
| Comfortable | -0.31 | -0.57 |  | 3.66 | 1.71 |
| Simple | -1.12 | 0.26 |  | 2.50 | 1.96 |
| Timid | -0.72 | 0.70 |  | 1.62 | 1.65 |
| Uncooperative | 11.16 | 3.12 |  | 0.44 | 1.00 |
| Odd | 3.55 | 2.02 |  | 0.78 | 1.37 |
| Dual dehumanization – UH ratings |  |  |  |  |  |
| Ambitious | -0.08 | -1.01 |  | 4.28 | 1.93 |
| Analytic | -0.46 | -0.84 |  | 4.18 | 1.96 |
| Imaginative | 0.13 | -1.17 |  | 4.42 | 1.98 |
| Passionate | -1.16 | -0.53 |  | 3.63 | 2.24 |
| High-strung | -1.39 | 0.27 |  | 2.53 | 2.24 |
| Frivolous | -0.44 | -0.89 |  | 4.16 | 2.04 |
| Irresponsible | -0.90 | -0.69 |  | 3.93 | 2.16 |
| Reserved | -1.09 | 0.33 |  | 2.50 | 2.07 |
| Broad-minded | 0.53 | -1.23 |  | 4.56 | 1.83 |
| Humble | -0.08 | -1.06 |  | 4.36 | 1.97 |
| Conscientious | -0.57 | -0.78 |  | 4.04 | 1.99 |
| Thorough | -1.26 | -0.19 |  | 3.21 | 2.14 |
| Disorganized | -1.00 | -0.58 |  | 3.80 | 2.12 |
| Ignorant | -1.44 | -0.15 |  | 3.18 | 2.28 |
| Rude | -0.77 | -0.73 |  | 4.06 | 2.07 |
| Suspicious | -1.05 | 0.53 |  | 2.09 | 2.09 |
| Active | -0.11 | 1.00 |  | 1.35 | 1.75 |
| Curious | -0.32 | 0.94 |  | 1.44 | 1.83 |
| Friendly | -0.82 | 0.67 |  | 1.73 | 1.91 |
| Artistic | 1.90 | -1.64 |  | 4.82 | 1.69 |
| Impatient | -1.19 | 0.42 |  | 2.15 | 2.07 |
| Impulsive | -0.95 | 0.57 |  | 2.05 | 2.04 |
| Jealous | -1.31 | 0.24 |  | 2.56 | 2.16 |
| Shy | -0.78 | 0.67 |  | 1.90 | 1.99 |
| Contented | -1.07 | 0.46 |  | 2.17 | 2.09 |
| Even-tempered | -1.00 | 0.36 |  | 2.34 | 2.02 |
| Relaxed | -0.65 | 0.70 |  | 1.70 | 1.84 |
| Comfortable | -0.81 | 0.65 |  | 1.87 | 2.00 |
| Simple | -0.32 | 0.83 |  | 1.77 | 1.89 |
| Timid | 0.17 | 1.03 |  | 1.41 | 1.71 |
| Uncooperative | -0.47 | 0.78 |  | 1.73 | 1.88 |
| Odd | -0.98 | 0.45 |  | 2.20 | 2.03 |
| Dual dehumanization – HN ratings |  |  |  |  |  |
| Ambitious | 1.21 | -1.21 |  | 4.84 | 1.34 |
| Analytic | -0.04 | -0.90 |  | 4.67 | 1.44 |
| Imaginative | 0.95 | -1.20 |  | 4.98 | 1.25 |
| Passionate | 1.30 | -1.24 |  | 4.90 | 1.33 |
| High-strung | -0.86 | -0.42 |  | 4.00 | 1.77 |
| Frivolous | -0.76 | -0.53 |  | 4.12 | 1.73 |
| Irresponsible | -0.94 | -0.49 |  | 4.03 | 1.85 |
| Reserved | -0.50 | -0.54 |  | 4.13 | 1.66 |
| Broad-minded | -0.33 | -0.74 |  | 4.40 | 1.62 |
| Humble | -1.00 | -0.43 |  | 4.19 | 1.68 |
| Conscientious | 0.36 | -0.94 |  | 4.64 | 1.45 |
| Thorough | -0.59 | -0.51 |  | 4.25 | 1.57 |
| Disorganised | -0.89 | -0.35 |  | 3.89 | 1.75 |
| Ignorant | -0.93 | -0.48 |  | 4.01 | 1.84 |
| Rude | -0.90 | -0.54 |  | 4.05 | 1.86 |
| Suspicious | -0.14 | -0.88 | 0.3% | 4.45 | 1.64 |
| Active | -0.07 | -0.74 |  | 4.58 | 1.37 |
| Curious | 3.09 | -1.63 |  | 5.05 | 1.25 |
| Friendly | 0.02 | -0.77 |  | 4.65 | 1.34 |
| Artistic | -0.06 | -0.96 |  | 4.73 | 1.48 |
| Impatient | -0.24 | -0.81 |  | 4.39 | 1.64 |
| Impulsive | -0.34 | -0.62 |  | 4.34 | 1.53 |
| Jealous | 0.39 | -1.03 |  | 4.57 | 1.58 |
| Shy | -0.92 | -0.39 |  | 4.05 | 1.72 |
| Contented | -0.91 | -0.36 |  | 4.22 | 1.58 |
| Even-tempered | -0.65 | -0.38 |  | 4.07 | 1.62 |
| Relaxed | -0.54 | -0.42 |  | 4.25 | 1.51 |
| Comfortable | -0.67 | -0.51 |  | 4.29 | 1.54 |
| Simple | -1.09 | -0.25 |  | 3.77 | 1.85 |
| Timid | -1.09 | -0.15 |  | 3.87 | 1.72 |
| Uncooperative | -1.06 | -0.37 |  | 3.89 | 1.88 |
| Odd | -1.15 | -0.29 |  | 3.76 | 1.89 |
| Dual dehumanization – Desirability ratings^*^ |  |  |  |  |  |
| Ambitious | 2.98 | -1.58 |  | 5.97 | 1.25 |
| Analytic | 0.71 | -0.90 | 0.3% | 5.55 | 1.35 |
| Imaginative | 2.88 | -1.62 |  | 6.13 | 1.17 |
| Passionate | 3.06 | -1.66 |  | 6.16 | 1.14 |
| High-strung | 2.29 | 1.65 |  | 1.94 | 1.35 |
| Frivolous | 1.06 | 1.32 |  | 2.11 | 1.42 |
| Irresponsible | 8.31 | 2.86 |  | 1.40 | 0.93 |
| Reserved | -0.29 | 0.21 |  | 3.58 | 1.62 |
| Broad-minded | 2.36 | -1.54 |  | 5.88 | 1.37 |
| Humble | 0.56 | -1.03 |  | 5.87 | 1.29 |
| Conscientious | 2.71 | -1.70 |  | 5.92 | 1.47 |
| Thorough | 3.02 | -1.68 | 0.3% | 5.98 | 1.29 |
| Disorganised | 6.53 | 2.40 |  | 1.53 | 0.99 |
| Ignorant | 9.68 | 2.98 |  | 1.45 | 1.03 |
| Rude | 9.76 | 2.89 |  | 1.42 | 0.92 |
| Suspicious | 1.05 | 1.24 |  | 2.12 | 1.37 |
| Active | 2.09 | -1.32 |  | 5.92 | 1.19 |
| Curious | 1.63 | -1.24 |  | 5.77 | 1.33 |
| Friendly | 4.58 | -1.95 |  | 6.33 | 1.03 |
| Artistic | 1.93 | -1.31 |  | 5.93 | 1.20 |
| Impatient | 6.94 | 2.44 |  | 1.66 | 1.13 |
| Impulsive | -0.32 | 0.74 |  | 2.54 | 1.55 |
| Jealous | 5.37 | 2.30 |  | 1.66 | 1.20 |
| Shy | 0.78 | 0.99 |  | 2.41 | 1.36 |
| Contented | 1.59 | -1.41 |  | 5.87 | 1.44 |
| Even-tempered | 2.98 | -1.72 |  | 5.97 | 1.37 |
| Relaxed | 0.22 | -1.03 |  | 6.18 | 0.99 |
| Comfortable | 4.63 | -1.98 |  | 6.24 | 1.12 |
| Simple | -0.91 | 0.17 |  | 3.66 | 1.86 |
| Timid | 0.60 | 1.06 |  | 2.34 | 1.47 |
| Uncooperative | 7.25 | 2.52 | 0.3% | 1.56 | 1.04 |
| Odd | 0.42 | 1.05 |  | 2.27 | 1.45 |
| Feeling thermometer *the protagonist’s* group^+^ | 2.28 | -1.54 |  | 79.61 | 22.88 |
| Blatant group dehumanization |  |  |  |  |  |
| Savage, aggressive | 4.37 | 2.16 |  | 1.70 | 1.32 |
| Backward, primitive | 3.24 | 1.92 |  | 1.84 | 1.41 |
| Lacking morals | 4.27 | 2.20 |  | 1.80 | 1.48 |
| Barbaric, cold-hearted | 5.84 | 2.44 |  | 1.63 | 1.27 |
| Refined and cultured^r^ | -0.42 | 0.59 |  | 3.16 | 1.69 |
| Rational and logical^r^ | 0.53 | 0.89 |  | 2.69 | 1.45 |
| Scientifically/technologically advanced^r^ | -0.80 | 0.25 |  | 3.78 | 1.81 |
| Capable of self-control^r^ | 1.11 | 1.34 |  | 2.42 | 1.65 |
| Mature, responsible^r^ | 0.95 | 1.24 |  | 2.40 | 1.55 |
| Empathic concern^x^ |  |  |  |  |  |
| I often have tender, concerned feelings for people less fortunate than me | 1.15 | -1.24 |  | 4.12 | 1.01 |
| Sometimes I don't feel very sorry for other people when they are having problems^r^ | -0.12 | -0.99 |  | 4.01 | 1.19 |
| When I see someone being taken advantage of, I feel kind of protective towards them | 1.47 | -1.30 |  | 4.20 | 0.95 |
| Other people's misfortunes do not usually disturb me a great deal^r^ | 0.88 | -1.26 |  | 4.12 | 1.09 |
| When I see someone being treated unfairly, I sometimes don't feel very much pity for them^r^ | 1.09 | -1.41 |  | 4.20 | 1.12 |
| I am often quite touched by things that I see happen | 0.66 | -1.09 | 0.3% | 4.06 | 1.03 |
| I would describe myself as a pretty soft-hearted person | 0.63 | -1.16 | 0.3% | 4.08 | 1.10 |

*Note.* ^d^ = distractor item, ^r^ = reverse scored. Participants were asked to indicate their agreement on scales ranging from 0 to 6, with the exception of the scales marked with ^*^ (= from 1-7), ^+^ (= from 0-100), and ^x^ (= from 1-5). The scales were presented in a fixed order whereas the items within the scales were presented in a random order. Missing values were imputed using the expectation-maximization algorithm in SPSS 24. Means and standard deviations are calculated after imputing missing values. HN = Human Nature, UH = Uniquely Human, CS = Communal Sharing.

**Table S3**

*Estimates for the retained measurement model in The Preliminary Study*

| Parameter | Unstandardized | SE | Standardized |
| --- | --- | --- | --- |
|  | Factor loadings | | |
| Kama muta labels factor |  |  |  |
| Heartwarming | 1 | 0 | 0.89 |
| Moved | 1.13 | 0.05 | 0.90 |
| Touched | 1.17 | 0.05 | 0.93 |
|  |  |  |  |
| Protagonist humanization factor |  |  |  |
| The protagonist strikes me as very human | 1 | 0 | 0.64 |
| The way the protagonist acts demonstrates what its really like to be human | 1.60 | 0.14 | 0.80 |
| The protagonist is an example of the most important parts of being human | 1.75 | 0.15 | 0.84 |
|  |  |  |  |
| Motivation for CS factor |  |  |  |
| The protagonist would "give the shirt off their back" for you | 1 | 0 | 0.82 |
| What is mine is yours is true for this relationship | 1.09 | 0.06 | 0.87 |
| You share food with this person | 0.85 | 0.06 | 0.75 |
| If this person needed help, you could cancel plans to give it | 0.95 | 0.05 | 0.86 |
| One for all and all for one is true for this relationship | 1.05 | 0.06 | 0.87 |
| What happens to this person is almost as important to you as what happens to you | 0.99 | 0.06 | 0.79 |
|  |  |  |  |
| Out-group feeling thermometer one-indicator factor | 1 | 0 | 0.98 |
|  |  |  |  |
| Blatant group dehumanization factor |  |  |  |
| Savage, aggressive | 1 | 0 | 0.64 |
| Backward, primitive | 1 | 0.09 | 0.60 |
| Lacking morals | 1.51 | 0.12 | 0.85 |
| Barbaric, cold hearted | 1.02 | 0.06 | 0.68 |
| Refined and cultured | 1.02 | 0.15 | 0.51 |
| Rational and logical | 1.09 | 0.14 | 0.63 |
| Scientifically/ technologically advanced | 0.65 | 0.15 | 0.30 |
| Capable of self-control | 1.24 | 0.15 | 0.63 |
| Mature and responsible | 1.32 | 0.15 | 0.72 |
|  |  |  |  |
|  | Factor covariances | | |
| Protagonist hum. – kama muta | 0.86 | 0.11 | 0.83 |
| Mot. for CS – kama muta | 1.24 | 0.16 | 0.60 |
| Mot. for CS – Protagonist hum. | 0.71 | 0.10 | 0.67 |
| OG feeling ther. – kama muta | 1.78 | 0.22 | 0.56 |
| OG feeling ther. – Protagonist hum. | 0.92 | 0.13 | 0.57 |
| OG feeling ther. – Mot. for CS | 1.70 | 0.23 | 0.53 |
| Blat. group dehum. – kama muta | -0.50 | 0.09 | -0.41 |
| Blat. group dehum. – Protagonist hum. | -0.29 | 0.06 | -0.47 |
| Blat. group dehum. – Mot. for CS | -0.55 | 0.10 | -0.45 |
| Blat. group dehum. – OG feeling ther. | -1.37 | 0.18 | -0.74 |

*Note.* CS = Communal Sharing, OG = Out-group.

**Table S4**

*Covariance matrix for retained model in The Preliminary Study*

**Table S5**

*Descriptive statistics for scales and items in Study 1*

| Scale | Kurtosis | Skew | Mean | SD |
| --- | --- | --- | --- | --- |
| Social dominance orientation |  |  |  |  |
| An ideal society requires some groups to be on top and others to be on the bottom | -0.90 | 0.60 | 2.88 | 1.88 |
| Some groups of people are simply inferior to other groups | -0.25 | 1.09 | 2.27 | 1.75 |
| No one group should dominate in society^r^ | 1.11 | 1.34 | 2.20 | 1.56 |
| Groups at the bottom are just as deserving as groups at the top^r^ | 0.81 | 1.25 | 2.23 | 1.50 |
| Group equality should not be our primary goal | -1.02 | 0.48 | 3.18 | 1.96 |
| It is unjust to try to make groups equal | -0.69 | 0.76 | 2.75 | 1.88 |
| We should do what we can to equalize conditions for different groups^r^ | 0.55 | 1.11 | 2.39 | 1.56 |
| We should work to give all groups an equal chance to succeed^r^ | 2.56 | 1.64 | 1.95 | 1.32 |
| Kama muta – physical sensations |  |  |  |  |
| Moist eyes or cried | -1.28 | 0.43 | 2.12 | 2.05 |
| Chills or goosebumps | -1.15 | 0.38 | 2.20 | 1.92 |
| Warm feeling in the chest | -0.80 | -0.49 | 3.59 | 1.87 |
| Kama muta – appraisals |  |  |  |  |
| I observed an incredible bond | 0.36 | -1.01 | 4.34 | 1.60 |
| … a special sense of belonging | -0.61 | -0.61 | 3.68 | 1.81 |
| … an exceptional sense of closeness appear | 0.39 | -0.93 | 4.15 | 1.58 |
| … the emergence of a remarkable feeling of oneness | -0.64 | -0.52 | 3.61 | 1.76 |
| … a unique kind of love spring up | -0.06 | -0.83 | 4.10 | 1.71 |
| … a phenomenal feeling of being appreciated | 0.14 | -0.87 | 4.17 | 1.63 |
| … an astonishing sense of someone needing a particular person or being needed by someone | 0.14 | -0.87 | 4.17 | 1.63 |
| … exceptional care being given to someone | -0.30 | -0.73 | 3.97 | 1.74 |
| Kama muta – emotion labels |  |  |  |  |
| It was heartwarming | 2.11 | -1.54 | 4.71 | 1.53 |
| I was moved | 0.07 | -0.95 | 4.21 | 1.73 |
| I was touched | 0.37 | -1.05 | 4.35 | 1.67 |
| I was anxious^d^ | 0.02 | 0.98 | 1.28 | 1.48 |
| Protagonist humanization |  |  |  |  |
| *The protagonist* seemed very human | 6.05 | -2.17 | 5.40 | 0.97 |
| T*he protagonist* actions demonstrate how human *the protagonist* is | 3.90 | -1.80 | 5.14 | 1.18 |
| *The protagonist* shows what being human truly is | 2.00 | -1.45 | 4.98 | 1.30 |
| Motivation to develop a CS relationship |  |  |  |  |
| *The protagonist* would ‘give the shirt of their back’ for you | 0.32 | -0.91 | 4.18 | 1.61 |
| ‘What is mine is yours’ is true for this relationship | 0.31 | -0.96 | 4.17 | 1.66 |
| You share food with this person | 2.04 | -1.53 | 4.79 | 1.51 |
| If this person needed help, you could cancel plans to give it | 1.58 | -1.28 | 4.50 | 1.46 |
| ‘One for all and all for one’ is true for this relationship | 0.10 | -0.88 | 4.06 | 1.70 |
| What happens to this person is almost as important to you as what happens to you | -0.66 | -0.56 | 3.67 | 1.81 |
| Connectedness of self to humanity^*^ | -0.39 | -0.33 | 4.65 | 1.52 |
| Connectedness of *the protagonist* to humanity^*^ | 1.19 | -1.25 | 5.83 | 1.34 |
| Feeling thermometer US Americans (in-group)^+^ | 0.08 | -0.76 | 76.88 | 20.65 |
| Feeling thermometer Black people^+^ | -0.12 | -0.75 | 70.70 | 25.83 |
| Feeling thermometer Pakistanis^+^ | -0.66 | -0.40 | 62.77 | 27.95 |
| Feeling thermometer Indians^+^ | -0.19 | -0.62 | 69.36 | 25.07 |
| Feeling thermometer Gay men^+^ | -0.25 | -0.75 | 68.45 | 28.36 |
| Feeling thermometer Thai people^+^ | -0.12 | -0.62 | 69.48 | 24.41 |
| Feeling thermometer *group presented* | 0.03 | -0.73 | 70.97 | 24.82 |
| Blatant dehumanization |  |  |  |  |
| Savage, aggressive | 1.11 | 1.32 | 2.01 | 1.31 |
| Backward, primitive | 0.51 | 1.18 | 2.17 | 1.49 |
| Lacking morals | 1.48 | 1.46 | 2.08 | 1.47 |
| Barbaric, cold-hearted | 1.76 | 1.51 | 1.85 | 1.24 |
| Refined and cultured^r^ | -0.24 | 0.40 | 3.04 | 1.44 |
| Rational and logical^r^ | -0.46 | 0.37 | 2.81 | 1.36 |
| Scientifically/technologically advanced^r^ | -0.52 | 0.22 | 3.39 | 1.56 |
| Capable of self-control^r^ | 0.37 | 0.89 | 2.40 | 1.35 |
| Mature, responsible^r^ | -0.04 | 0.59 | 2.70 | 1.37 |

*Note.* ^d^ = distractor item, ^r^ = reverse scored. Participants were asked to indicate their agreement on scales ranging from 0 to 6, with the exception of the scales marked with ^*^ (= from 1-7) and ^+^ (= from 0-100). The scales were presented in a fixed order whereas the items within the scales were presented in a random order. There were no missing values in Study 1. CS = Communal Sharing.

**Table S6**

*Estimates for the retained measurement model in Study 1*

| Parameter | Unstandardized | SE | Standardized |
| --- | --- | --- | --- |
|  | Factor loadings | | |
| Kama muta labels factor |  |  |  |
| Heartwarming | 1.00 | 0.00 | 0.88 |
| Moved | 1.21 | 0.04 | 0.94 |
| Touched | 1.20 | 0.04 | 0.97 |
|  |  |  |  |
| Protagonist humanization factor |  |  |  |
| The protagonist seemed very human | 1.00 | 0.00 | 0.84 |
| The way the protagonist’s actions demonstrate how human the protagonist is | 1.20 | 0.07 | 0.83 |
| The protagonist shows what being human truly is | 1.35 | 0.08 | 0.84 |
|  |  |  |  |
| Motivation for CS factor |  |  |  |
| The protagonist would "give the shirt off their back" for you | 1.00 | 0.00 | 0.83 |
| What is mine is yours is true for this relationship | 1.11 | 0.05 | 0.89 |
| You share food with this person | 0.75 | 0.05 | 0.66 |
| If this person needed help, you could cancel plans to give it | 0.88 | 0.05 | 0.80 |
| One for all and all for one is true for this relationship | 1.00 | 0.06 | 0.79 |
| What happens to this person is almost as important to you as what happens to you | 1.11 | 0.06 | 0.82 |
|  |  |  |  |
| Out-group feeling thermometer one-indicator factor | 1.00 | 0.00 | 0.98 |
|  |  |  |  |
| Blatant group dehumanization factor |  |  |  |
| Savage, aggressive | 1.00 | 0.00 | 0.62 |
| Backward, primitive | 1.00 | 0.08 | 0.54 |
| Lacking morals | 0.96 | 0.08 | 0.53 |
| Barbaric, cold hearted | 0.86 | 0.06 | 0.56 |
| Refined and cultured | 1.43 | 0.15 | 0.80 |
| Rational and logical | 1.33 | 0.14 | 0.79 |
| Scientifically/ technologically advanced | 1.34 | 0.16 | 0.72 |
| Capable of self-control | 1.12 | 0.13 | 0.67 |
| Mature and responsible | 1.43 | 0.15 | 0.84 |
|  |  |  |  |
|  | Factor covariances | | |
| Protagonist hum. – kama muta | 0.66 | 0.07 | 0.61 |
| Mot. for CS – kama muta | 1.41 | 0.12 | 0.64 |
| Mot. for CS – Protagonist hum. | 0.62 | 0.07 | 0.57 |
| OG feeling ther. – kama muta | 1.02 | 0.18 | 0.31 |
| OG feeling ther. – Protagonist hum. | 0.68 | 0.12 | 0.34 |
| OG feeling ther. – Mot. for CS | 1.05 | 0.18 | 0.32 |
| Blat. group dehum. – kama muta | -0.31 | 0.07 | -0.29 |
| Blat. group dehum. – Protagonist hum. | -0.19 | 0.05 | -0.29 |
| Blat. group dehum. – Mot. for CS | -0.36 | 0.07 | -0.33 |
| Blat. group dehum. – OG feeling ther. | -1.31 | 0.17 | -0.67 |

*Note.* CS = Communal Sharing, OG = Out-group.

**Table S7**

*Covariance matrix for replicated model in Study 1*

**Table S8**

*Covariance matrix for model with SDO as control variable in Study 1*

**Table S9**

*Results from video pre-test in Study 2*

|  |  | Kama muta | | | Amusement | | |
| --- | --- | --- | --- | --- | --- | --- | --- |
|  |  |  | Physiology | Labels |  | Physiology | Labels |
| Video | Emotion (group) | n | M(*SD*) | M(*SD*) | n | M(*SD*) | M(*SD*) |
| **Oprah** | KM (Black) | 37 | 1.72(1.45) | 3.14(1.72) | 37 | 0.49(1.07) | 0.73(1.34) |
| **Wedding** | KM (Black) | 37 | 1.67(1.59) | 2.71(2.03) | 37 | 0.46(0.90) | 0.85(1.24) |
| Talent Show | KM (Black) | 37 | 1.78(1.85) | 2.48(2.03) | 37 | 0.68(1.31) | 1.14(1.50) |
| **Soldier Homecoming** | KM (Gay) | 37 | 2.73(1.83) | 4.13(1.70) | 37 | 1.49(2.02) | 1.57(1.86) |
| **Colorblind** | KM (Gay) | 37 | 2.21(1.86) | 3.39(2.10) | 37 | 2.35(2.26) | 2.65(1.83) |
| **Proposal** | KM (Gay) | 37 | 2.20(1.80) | 3.18(2.03) | 37 | 2.14(1.89) | 2.47(1.62) |
| **Stand-up** | AM (Black) | 35 | 0.73(1.06) | 0.76(1.16) | 35 | 3.54(2.08) | 3.96(1.78) |
| **Sketch** | AM (Black) | 35 | 0.84(1.16) | 1.04(1.44) | 35 | 3.17(1.92) | 3.44(1.90) |
| **Black-ish** | AM (Black) | 35 | 0.42(0.86) | 0.68(0.97) | 35 | 2.37(1.94) | 2.40(1.65) |
| **Prank** | AM (Gay) | 35 | 0.48(0.81) | 0.61(1.14) | 35 | 2.74(1.69) | 3.00(1.84) |
| Slapstick | AM (Gay) | 35 | 0.74(1.02) | 0.77(1.24) | 35 | 1.74(1.79) | 2.16(1.85) |
| **Modern Family** | AM (Gay) | 35 | 0.53(0.92) | 0.90(1.35) | 35 | 2.77(1.70) | 3.06(1.58) |

*Note.* KM = kama muta. AM = Amusement. Videos highlighted in bold were selected for the main study.

**Table S10**

*Test of H1 in Study 2: Kama muta predicts protagonist humanization*

| Parameter | DF1 | DF2 | *F* | *p* |
| --- | --- | --- | --- | --- |
| Intercept | 1 | 219 | 5439.65 | <.001 |
| Time | 1 | 659 | .12 | .729 |
| Group | 1 | 659 | .13 | .723 |
| Content | 1 | 659 | 148.32 | <.001 |
| Time*Content | 1 | 659 | 34.87 | <.001 |
| Group*Content | 1 | 219 | 6.97 | .009 |

**Table S11**

*Covariance matrix for within-participant model in Study 2*

*Note.* CSDiff = Difference score between motivation for CS after kama muta video and after amusing video. FTDIFF = Diffrence score for Feeling thermometer toward out-group. BLATDIFF = Difference score between blatant group dehumanization. HUMDIFF = Difference score between protagonist humanization. HUMMEANC = Grand mean centered values for protagonist humanization. CSMEANC = Grand mean centered values for motivation for CS. FTMEANC = Grand mean values for feeling thermometer toward out-group.

**Table S12**

*Univariate normality of variables in within-participant model in Study 2*

| Variable | Skewness | Kurtosis |
| --- | --- | --- |
| CSDIFF | 0.91 | 0.529 |
| FTDIFF | 0.497 | 8.352 |
| BLATDIFF | -0.569 | 6.1 |
| BEIDIFF | 0.845 | 0.261 |
| BEIMEANC | -0.759 | 0.221 |
| CSMEANC | -0.272 | -0.022 |
| FTMEANC | -0.319 | -0.946 |

Note. CSDiff = Difference score between motivation for CS after kama muta video and after amusing video. FTDIFF = Diffrence score for Feeling thermometer toward out-group. BLATDIFF = Difference score between blatant group dehumanization. HUMDIFF = Difference score between protagonist humanization. HUMMEANC = Grand mean centered values for protagonist humanization. CSMEANC =Grand mean centered values for motivation for CS. FTMEANC = Grand mean values for feeling thermometer toward out-group.

**Table S13**

*Ratings of protagonist humanization in Study 2 while controlling for blatant group dehumanization*

| Video content | Time | -1 SD | Mean | +1 SD | |
| --- | --- | --- | --- | --- | --- |
|  |  | Blatant group dehumanization at Time 1 as covariate | | |  |
| Moving | 1 | 5.15 [4.95, 5.34] | 4.81 [4.67, 4.95] | 4.47 [4.27, 4.66] | |
|  | 2 | 5.60 [5.42, 5.78] | 5.25 [5.12, 5.38] | 4.87 [4.71, 5.08] | |
| Funny | 1 | 4.71 [4.51, 4.93] | 4.35 [4.21, 4.50] | 3.99 [3.78, 4.20] | |
|  | 2 | 4.41 [4.09, 4.73] | 3.96 [3.73, 4.19] | 3.51 [3.19, 3.83] | |
|  |  | Blatant group dehumanization at Time 2 as covariate | | |  |
| Moving | 1 | 5.13 [4.93, 5.32] | 4.81 [4.67, 4.95] | 4.49 [4.29, 4.69] | |
|  | 2 | 5.59 [5.40, 5.77] | 5.25 [5.12, 5.38] | 4.91 [4.73, 5.09] | |
| Funny | 1 | 4.61 [4.40, 4.82] | 4.35 [4.20, 4.51] | 4.10 [3.88, 4.31] | |
|  | 2 | 4.50 [4.18, 4.82] | 3.96 [3.73, 4.18] | 3.42 [3.10, 3.74] | |

*Note.* Brackets show 95% confidence intervals. The values indicated correspond to the estimated means of protagonist humanization at different levels of blatant group dehumanization ratings at Time 1 and Time 2: -1SD, Mean and +1SD of their respective distributions.

**Table S14**

*SDO results in Study 2: SDO moderating the effect of kama muta on change in protagonist humanization from Time 1 to Time 2*

| Parameter | DF1 | DF2 | *F* | *p* |
| --- | --- | --- | --- | --- |
| Intercept | 1 | 218 | 1495.77 | <.001 |
| Time | 1 | 654 | .13 | .719 |
| Order | 1 | 654 | 9.28 | .002 |
| Content | 1 | 654 | 154.94 | <.001 |
| Group | 1 | 654 | 3.13 | .078 |
| SDO | 1 | 218 | 27.41 | <.001 |
| Time*Content | 1 | 654 | 35.28 | <.001 |
| Order*SDO | 1 | 654 | 11.66 | .001 |
| Group*SDO | 1 | 654 | 4.87 | .028 |
| Time*Order*Content | 3 | 562.19 | .66 | .577 |

**Table S15**

*RWA results in Study 2: RWA moderating the effect of kama muta on change in protagonist humanization from Time 1 to Time 2*

| Parameter | DF1 | DF2 | *F* | *p* |
| --- | --- | --- | --- | --- |
| Intercept | 1 | 219 | 793.56 | <.001 |
| Time | 1 | 657 | .12 | .728 |
| Order | 1 | 657 | .23 | .631 |
| Content | 1 | 657 | 142.07 | <.001 |
| Group | 1 | 657 | 6.62 | .010 |
| RWA | 1 | 219 | 2.11 | .148 |
| Time*Content | 1 | 657 | 35.21 | <.001 |
| Group*RWA | 1 | 657 | 8.42 | .004 |

**Supplemental Figures**


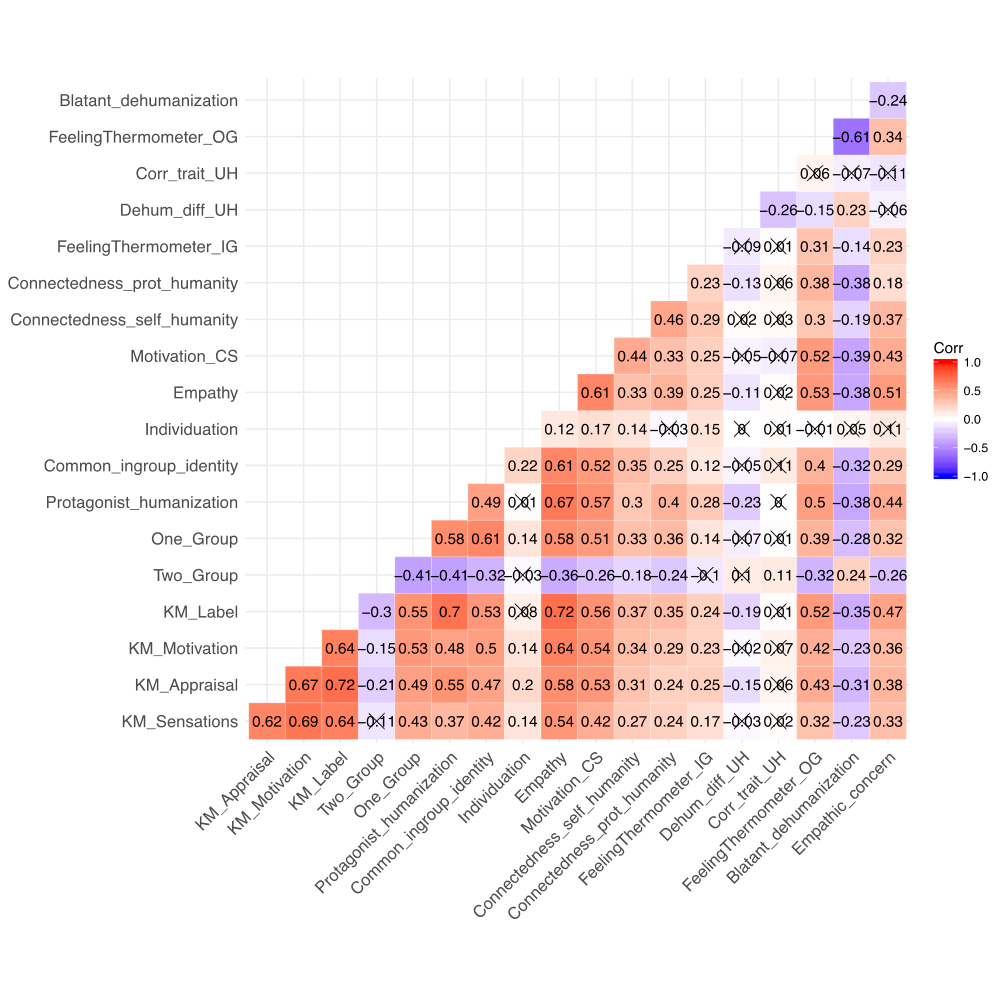


*Figure S1.* Correlation matrix of all composite values in the Preliminary Study.

*Note.* Crossed values are not significant at *p* < .05. Corr_trait_UH is the within-participants correlations between the attribution of traits and the UH ratings of these traits. Dehum_diff_UH is the difference score in attribution of high and low UH traits


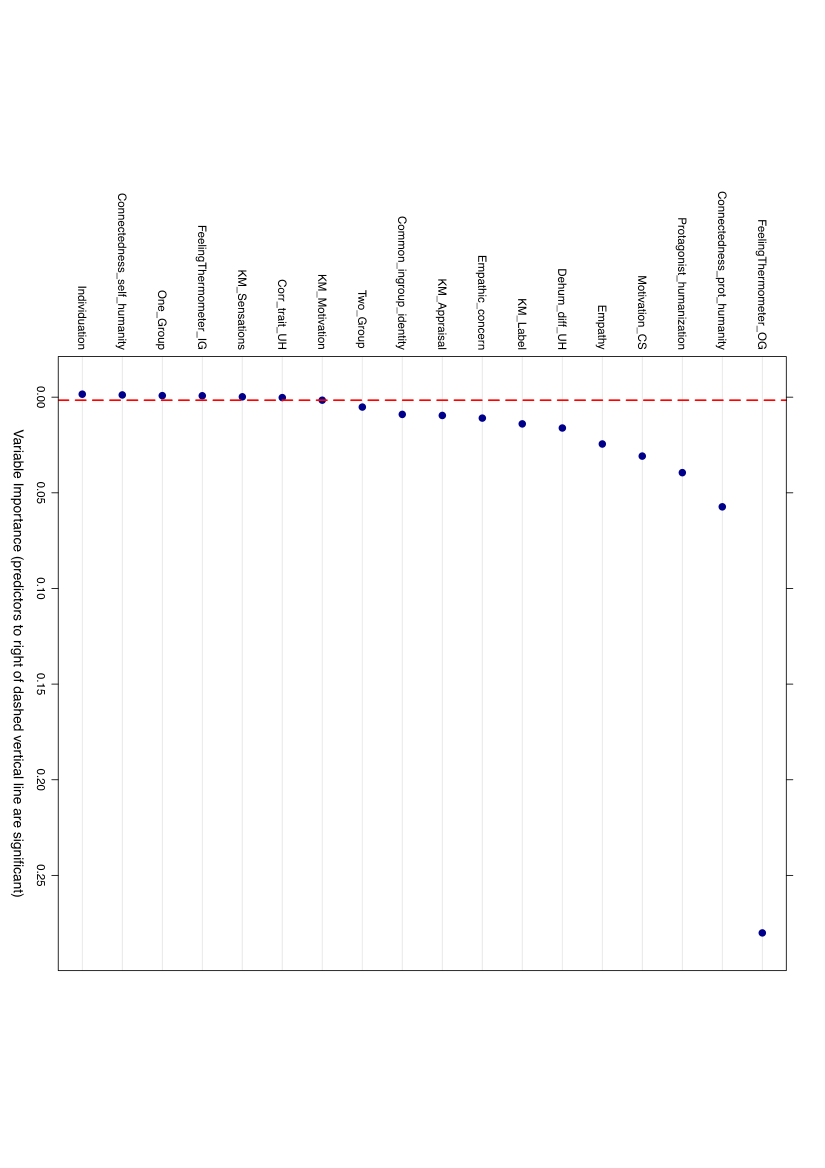


*Figure S2.* Permutation importance rankings with blatant group dehumanization as dependent variable from the Preliminary Study.

*Note.* Corr_trait_UH is the within-participants correlations between the attribution of traits and the UH ratings of these traits. Dehum_diff_UH is the difference score in attribution of high and low UH traits.


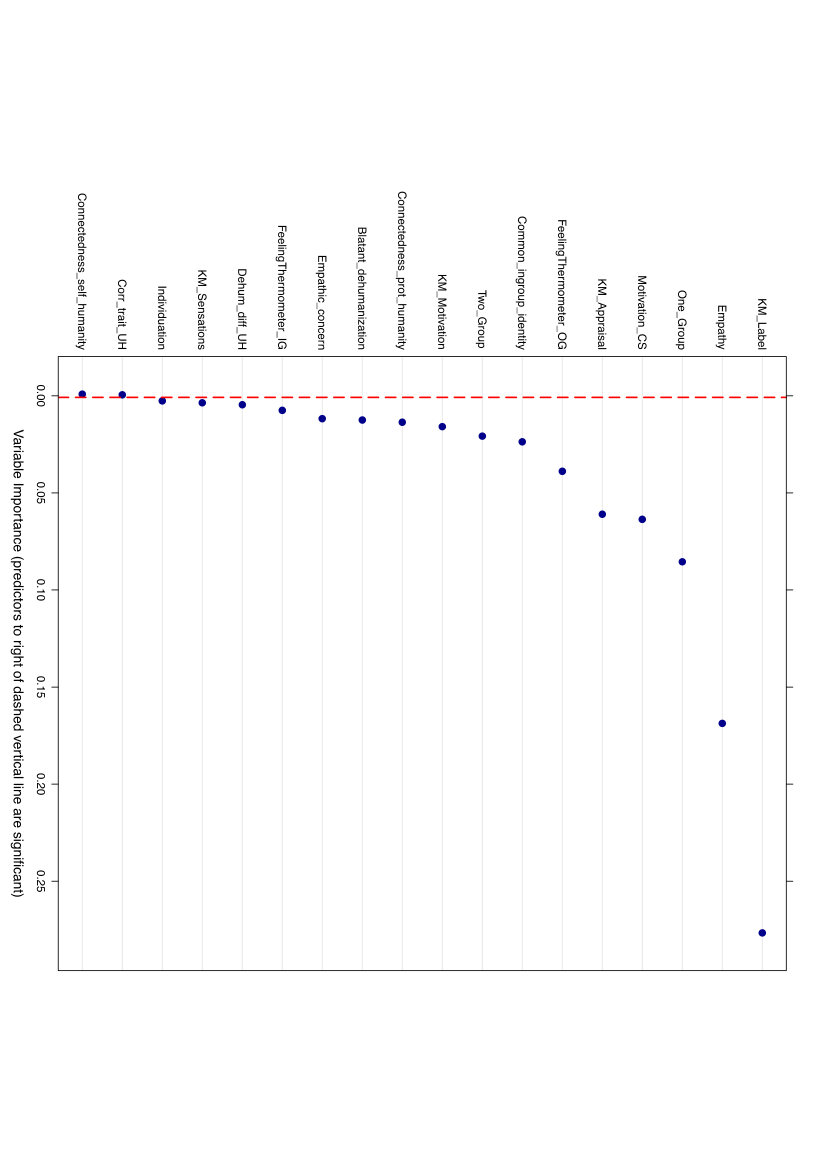


*Figure S3.* Permutation importance rankings with protagonist humanization as dependent variable from the Preliminary Study.

*Note.* Corr_trait_UH is the within-participants correlations between the attribution of traits and the UH ratings of these traits. Dehum_diff_UH is the difference score in attribution of high and low UH traits.


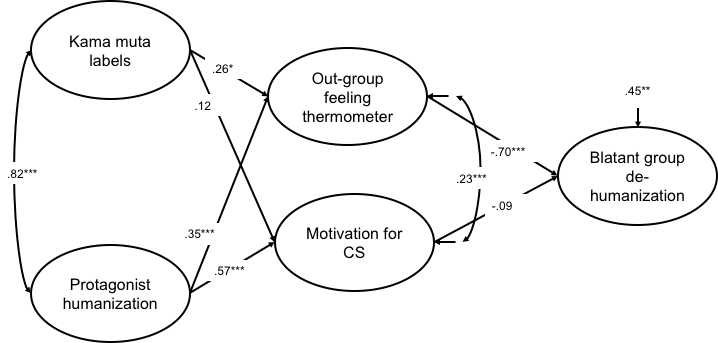


*Figure S4.* Latent factor model from the Preliminary Study with standardized estimates.

*Note.* * *p* < .05, ** *p* < .01, *** *p* < .001

**
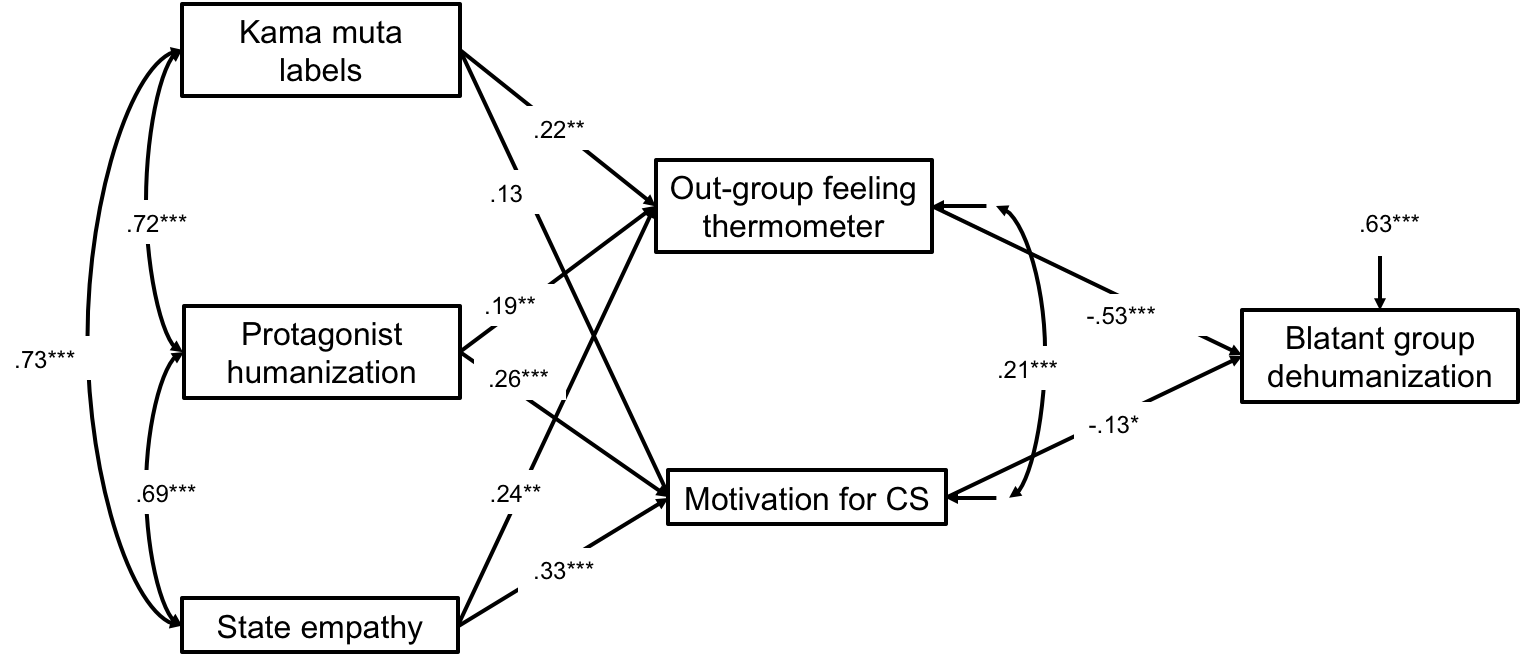
**

*Figure S5.* Path model from the Preliminary Study with state empathy as third IV, with standardized estimates.

*Note.* * *p* < .05, ** *p* < .01, *** *p* < .001


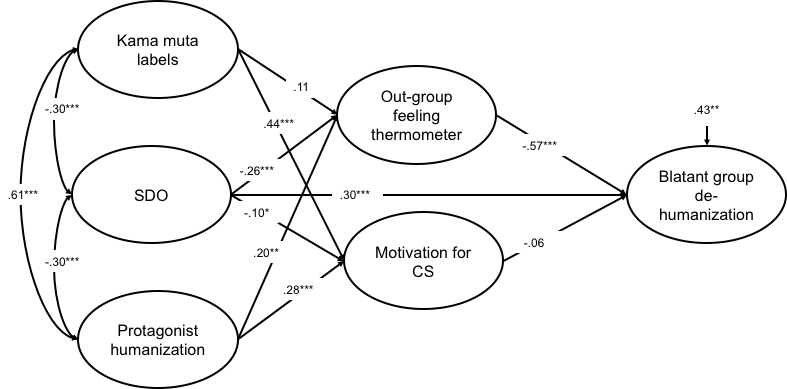


*Figure S6.* Latent factor model from Study 1 with SDO as a control variable, with standardized estimates.

*Note.* * *p* < .05, ** *p* < .01, *** *p* < .001

1. Random forest modeling is a type of supervised machine learning that relies on a forest of decision tress, hence the name. The data patterns are derived by an outcome variable, which makes this supervised machine learning. The decision trees, also called classification trees, are recursively partitioned where observations of similar response values are grouped, therefore measuring the relative importance of predictor variables by looking at how early during the portioning the variable is used. Several of these trees are then grown based on bootstrap samples, and for each tree a random subset of predictor variables is selected, resulting in a diverse set of trees which then are combined to evaluate relative variable importance (for a more in-depth discussion see IJzerman et al., 2016; Strobl, Malley, & Tutz, 2009). [↑](#footnote-ref-1)
